# Cryo‐Electron Microscopy for Unveiling the Sensitive Battery Materials

**DOI:** 10.1002/smsc.202100055

**Published:** 2021-09-15

**Authors:** Zhijin Ju, Huadong Yuan, Ouwei Sheng, Tiefeng Liu, Jianwei Nai, Yao Wang, Yujing Liu, Xinyong Tao

**Affiliations:** ^1^ College of Materials Science and Engineering Zhejiang University of Technology Hangzhou 310014 China

**Keywords:** battery materials, cryo-electron microscopy, electrolyte interphase, lithium metal anode, lithium metal batteries

## Abstract

Deep chemical and structural investigation of battery components is increasingly imperative for exploring new electrode materials and their performance iterations for the next‐generation of energy storage devices with high energy density. This is particularly true in the research realm of lithium (Li) metal and its derivatives for the robust anode. Conventionally, both Li metal and its solid electrolyte interphase (SEI) layer are chemically reactive and sensitive to electron‐beam irradiation, making the high‐resolution observation difficult to perform at native environment. Recently, the emergence of cryo‐electron microscopy (EM) has brought great opportunities to reveal the physicochemical properties of these energy materials. By means of cryo‐EM, the high‐resolution imaging of the samples at the nanometer or even atomic scale while maintaining their native state can be realized. Herein, the contributions of cryo‐EM to the characterization of sensitive battery materials are focused on, which are tentatively classified as the following: the visualization of Li dendrites, inactive Li, and the discussion regarding electrode interface chemistry. The review concludes by providing several proposals for the development of cryo‐EM in the future. It is hoped that this work will shed light on the in‐depth understanding of battery materials for high‐performance rechargeable batteries.

## Graphical abstract

The emergence of cryo‐electron microscopy has brought great opportunities to reveal the physicochemical properties of battery materials. Herein, the contributions of cryo‐electron microscopy to the characterization of sensitive battery materials are focused on. It is hoped that this review will shed light on the future directions for future research and promote the development of high‐performance rechargeable batteries.

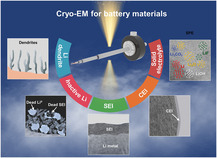

## 1 Introduction

The increasing demand for advanced electric vehicles and grid energy storage requires the optimization of current battery materials.^[^ 1, 2, 3, 4, 5 ^]^ The advanced battery systems paired with high specific capacity anode (alkali metal, silicon [Si]) and cathode (sulfur [S], air) hold great promise for current innovations in energy storage technology.^[^ 6, 7, 8, 9 ^]^ Especially, the Li metal batteries (LMBs) including Li—S and Li–air systems can improve the specific energy to ≈650 and ≈950 Wh kg^−1^, respectively, which are significantly higher than the state‐of‐the‐art Li‐ion batteries (≈250 Wh kg^−1^).^[^ 10, 11 ^]^ However, the commercialization of these battery materials are seriously impeded by their inherent challenges, such as dendrite growth on Li anode, serious volume expansion of Si anode, the shuttle effect in Li—S cells, and the oxygen crossover in Li–air cells.^[^ 12, 13, 14, 15, 16 ^]^

Currently, numerous efforts have been devoted to the search of viable solutions to aforementioned challenges. The fruitful reviews have summarized the progress of morphological designs, novel electrolyte formula, and artificial protective layer.^[^ 17, 18, 19, 20, 21 ^]^ However, less works focus on the exploration of electrochemical mechanism. However, deep chemical and structural investigation of battery components is increasingly imperative for exploring new electrode materials and their performance iterations for the next‐generation of energy storage devices with high energy density.^[^ 22, 23, 24 ^]^ Regretfully, these battery materials are always unstable when exposed to electron beam. For example, in the case of Li metal and S cathode, they will be easily destroyed due to low melting point under the strong electron beam radiation. In addition, the reaction intermediates, such as Li (poly)sulfides, Li—Si alloy, and Li peroxide, are very flimsy and it is difficult to explore their structural properties.^[^ 25, 26 ^]^ In addition, both the solid electrolyte interphase (SEI) and cathode electrolyte interphase (CEI) on electrode surface are in a metastable state, and significantly affected by the air and electron beam.^[^ ^27^ ^]^ Although a great deal of research has been done to understand their physical and chemical properties,^[^ 15, 28 ^]^ it is still challenging to directly observe the interface structure and gather the intrinsic information of electrode materials, especially at the nanoscale.

Cryo‐electron microscopy (cryo‐EM) has been awarded the 2017 Nobel Prize in Chemistry for its ability to achieve high‐resolution structural imaging of biomolecules.^[^ ^29^ ^]^ In the past few decades, cryo‐EM has gradually become an important research tool in structural biology.^[^ ^30^ ^]^ This technique has simplified and improved the imaging of biomolecules, which has brought biochemistry into a new era. At the same time, with the validity of cryo‐EM to obverse sensitive battery materials and realize the atomic structure resolution of Li metal and its interfaces in 2017,^[^ ^31^ ^]^ cryo‐EM shows great promise in the understanding of energy materials. At cryogenic temperatures, fragile battery materials can retain their original state and structure from electron beam damage, and also can be imaged at the micro/nanoscale, or even at the atomic scale.^[^ ^32^ ^]^ In recent years, the studies have increasingly focused on the electrochemical mechanism of electrode materials revealed by cryo‐EM techniques.^[^ 33, 34, 35, 36, 37, 38, 39, 40 ^]^ These new discoveries and breakthroughs have broadened our vision of battery material design and provided significant basis for solving the core problems in the energy field.

**Figure 1:**
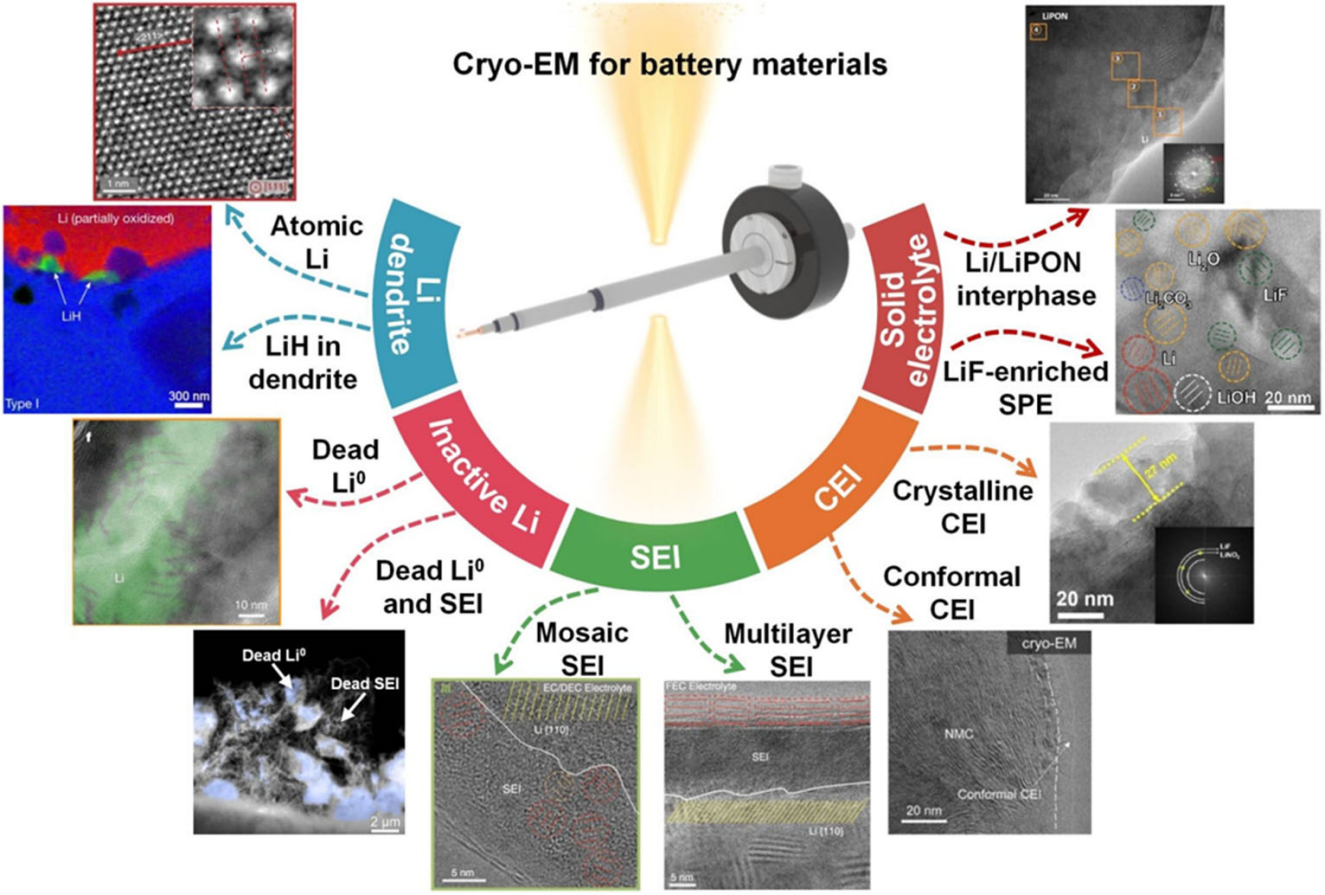
Cryo‐EM for battery materials. Part I, the atomic‐resolution observation and structural/chemical mapping of Li dendrites. (Top image) Reproduced with permission.^[^ ^31^ ^]^ Copyright 2017, AAAS. (Bottom image) Reproduced with permission.^[^ ^33^ ^]^ Copyright 2018, Springer Nature. Part II, the visualization and quantification of inactive Li. (Top image) Reproduced with permission.^[^ ^50^ ^]^ Copyright 2019, Springer Nature. (Bottom image). Reproduced with permission.^[^ ^57^ ^]^ Copyright 2021, Springer Nature. Part III, the structure and composition analysis of SEI layer on Li anode. (Mosaic and Multilayer SEI images) Reproduced with permission.^[^ ^31^ ^]^ Copyright 2017, AAAS. Part IV, the capture and identification of CEI film on cathode. (Bottom image) Reproduced with permission.^[^ ^114^ ^]^ Copyright 2021, Elsevier. (Top image) Reproduced with permission.^[^ ^115^ ^]^ Copyright 2019, Elsevier. Part V, the interface presentation of solid electrolyte at atomic scale. (Top image) Reproduced with permission.^[^ ^123^ ^]^ Copyright 2020, Elsevier. (Bottom image) Reproduced with permission.^[^ ^124^ ^]^ Copyright 2020, Wiley‐VCH.

Herein, we review the recent progress of cryo‐EM characterization on battery materials, and illustrate with case studies how cryo‐EM promotes the microstructural analysis and rational design of battery materials. First of all, as the generation of Li dendrite can result in the degradation of battery performance, the thorough understanding of its structure and composition will help us to fundamentally inhibit the dendrite formation. In addition, as the main culprit for the decaying battery efficiency, the physical and chemical properties of inactive Li are still poorly understood. Because the interface properties of the electrodes, including SEI and CEI, significantly affect the stability of the battery, a detailed analysis of them is also critical for the construction of high‐performance batteries. Especially for solid electrolyte, the elucidating of their nanostructure with Li metal is of guiding significance for comprehensive understanding battery performance and failure mechanism. Therefore, as shown in **Figure** **1**, we first discuss the atomic‐resolution observation and structural/chemical mapping of Li metal dendrites. Then, the typical inactive Li responsible for performance decay is visualized and quantified in virtue of cryo‐EM. In addition, the structure and composition analysis of SEI layer on Li anode is described. Subsequently, the structure of CEI film on cathode is also fully correlated with its function. Furthermore, the sensitive interface of solid electrolyte is investigated and compared through examples at atomic scale. Finally, some suggestions on how to further promote the development of high‐performance battery systems by cryo‐EM were also put forward.

## 2 Nanoscale Visualization of Li Metal Dendrites

The battery materials used in practical applications are macroscopic, while the inner operation mechanism of the battery often involves their microscopic properties. Thus, it is imperative to fully understand the characteristics of these materials at the micro‐, nanometer, and even atomic scales.^[^ 41, 42, 43 ^]^ Although the current characterization techniques have made us recognize that the formation of Li dendrites is to blame for the failure of LMBs,^[^ 11, 44, 45 ^]^ there is insufficient knowledge about the microstructure and composition of Li dendrites up to now. Therefore, it is extremely difficult to study their structural characteristics, especially at the atomic scale.

However, the conventional EM is used at room temperature and the high‐power electron beam can damage the native structure of electrode materials. In particular, the Li metal is highly sensitive to temperature and deformation rate,^[^ 46, 47 ^]^ which affect the physicochemical properties of investigated samples after continuous irradiation. By comparison, the cryo‐EM by using liquid nitrogen cooling can retain the sample temperature below −170 °C and significantly reduce the thermal damage to the sample during high‐resolution imaging.^[^ ^48^ ^]^ With the introduction of cryo‐EM into the research realm of energy materials, it is found that these sensitive battery materials including Li metal dendrites can be well protected and have good stability under the irradiation of electron beam, which provides a great opportunity to further study their microscopic properties.^[^ ^49^ ^]^

**Figure 2:**
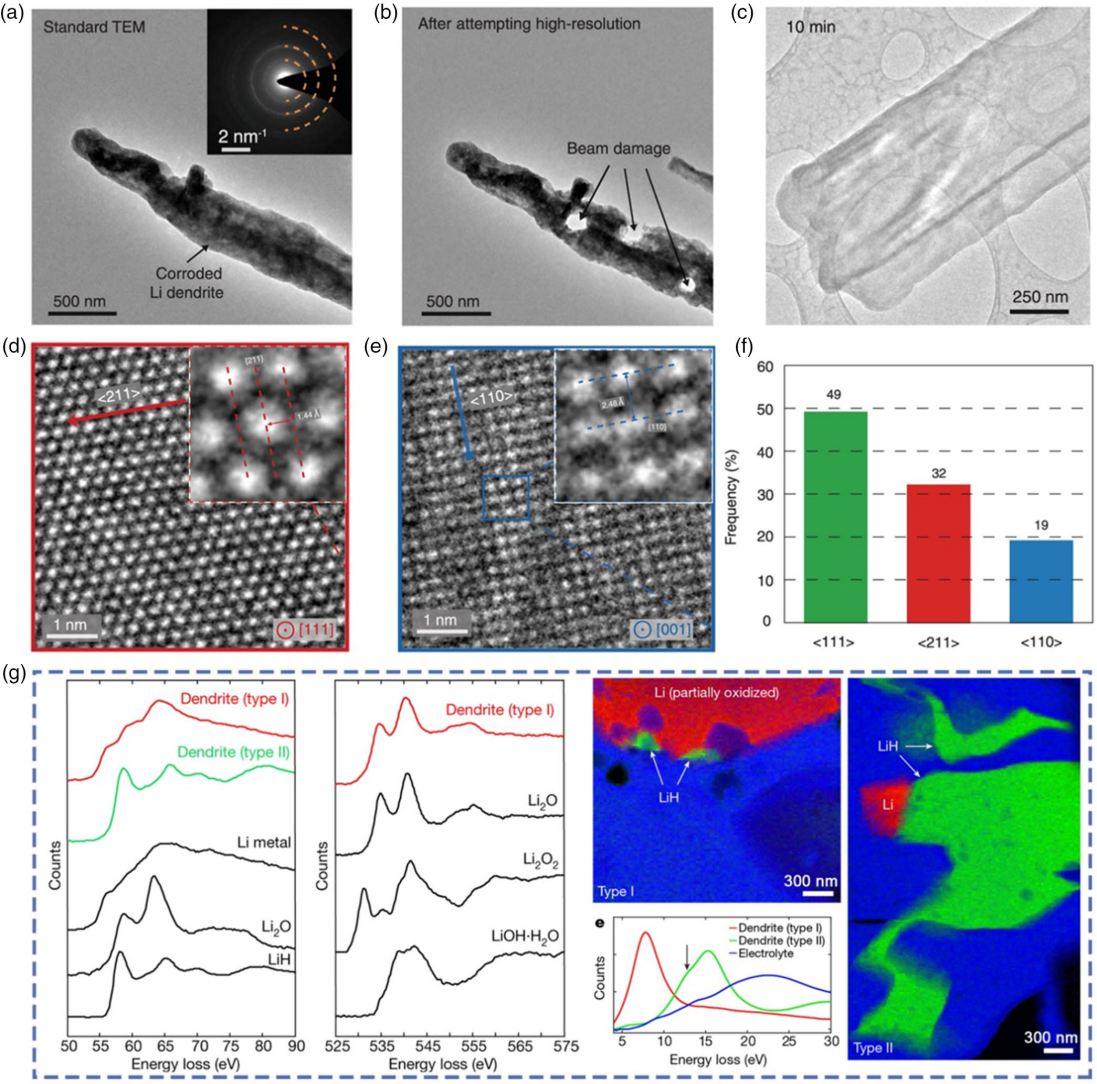
a) Standard TEM image of Li dendrite with ≈1 s air exposure at room temperature. b) TEM image of the damaged Li dendrite after exposure to electron beam. c) Cryo‐TEM image of Li dendrite after continuous electron‐beam irradiation for 10 min. d,e) Cryo‐TEM images of Li dendrites at atomic resolution along the (d) [111] and (e) [100] zone axis. f) Statistics showing preferred growth direction of Li dendrites is along <111>. a‐f) Reproduced with permission.^[^ ^31^ ^]^ Copyright 2017, AAAS. g) Determination and mapping of dendrite composition based on cryo‐STEM EELS. Reproduced with permission.^[^ ^33^ ^]^ Copyright 2018, Springer Nature.

Demonstrably, Cui and coworkers observe that Li dendrite is easily corroded by air during sample transfer under conventional transmission electron microscope (TEM) (**Figure** **2**a).^[^ ^31^ ^]^ The dendrite surface is very rough, and its selected area electron diffraction (SAED) is polycrystalline, corresponding to Li hydroxide. In adition, due to low melting point and weak atom bonding, dendritic Li is very unstable under the electron beam and its structure can be easily destroyed (Figure 2b). In contrast, Li dendrite can maintain its original structure with no change in morphology after long time irradiation at cryogenic temperature (Figure 2c). In addition, Cui et al. have pioneered atomic‐resolution observations of Li metal with the help of cryo‐TEM. As shown in Figure 2 d,e, individual Li atoms can be resolved from the high‐resolution transmission electron microscope (HRTEM) images of Li metal dendrites, suggesting that Li dendrites are single crystalline. These dendrites grow along <211> and<110> directions, respectively. The investigation about the growth behavior of Li dendrites shows that there are three main growth directions, corresponding to <111>, <110>, and <211>, respectively. The statistics (Figure 2f) reveal that Li dendrites in the growth direction of <111> occupy 49%, higher than that of <211> (32%) and <110> (19%). This phenomenon is because that Li dendrite prefers exposing the most densely packed {110} planes as the side surface to reduce the surface energy.^[^ ^31^ ^]^ Furthermore, it shows that the kinked dendrite in the carbonate electrolyte will change their direction of crystallization growth. The dendrite is shown to switch between the <211> and <110> growth directions with no crystal defects at these kinked regions. And the formation of kinked Li dendrite may be ascribed to changes in SEI composition and/or structure.

As a complement, the element distribution and content of the materials can be determined by combining energy dispersive X‐ray spectroscopy (EDS) and the electron energy loss spectrometer (EELS) equipped in cryo‐TEM, which can further provide the chemical composition information.^[^ ^49^ ^]^ Especially for some amorphous phases, EDS and EELS can be used to distinguish various components, explore the evolution of the interface, and comprehensively obtain the structural properties of materials. With the help of cryo‐STEM, Kourkoutis et al. studied the structure of solid–liquid interface in LMBs and mapped the structural and chemical of the interface (Figure 2g).^[^ ^33^ ^]^ Cryo‐STEM showed that there were two kinds of dendrites with different structure and composition on the Li anode. Type I dendrite is about 5 μm in diameter with low curvature and has extended SEI layer. The type II dendrite is generally hundreds of nanometers thick. The fine structures of the Li and O K‐edge show that the type I dendrite is composed primarily of Li metal and partial Li_2_O. However, the type II dendrite is surprisingly dominated by pure Li hydride (LiH). The low‐loss EELS mapping further demonstrates the partial oxidation of Li in type I dendrite, and the type II dendrite made from LiH. They suggest that the type II dendrite may be more likely to disconnect with electrodes during cycling, which causes the formation of dead Li and disproportionately capacity attenuation of battery. These results provide a new approach for the visualization of Li metal anode and stimulate researchers’ great interest in the observation of energy materials using cryo‐EM.

## 3 The Imaging of Inactive Li

The low Coulombic efficiency (CE) of LMBs is mainly due to the formation of inactive Li, also known as “dead Li,” which consists of Li components in useless SEI (dead SEI) and electrically isolated unreacted Li (dead Li^0^).^[^ 50, 51, 52 ^]^ Specifically, large volume expansion during Li deposition causes SEI destruction and regeneration. Repeated cycling will aggravate the inhomogeneity of SEI, which leads to the nonuniform Li stripping. And the insufficiently stripped Li will evolve into dead Li^0^ when losing electronic contact with the electrode.^[^ ^53^ ^]^ The formation of inactive Li will hinder the diffusion of Li ions and finally result in the capacity degradation of battery.

**Figure 3:**
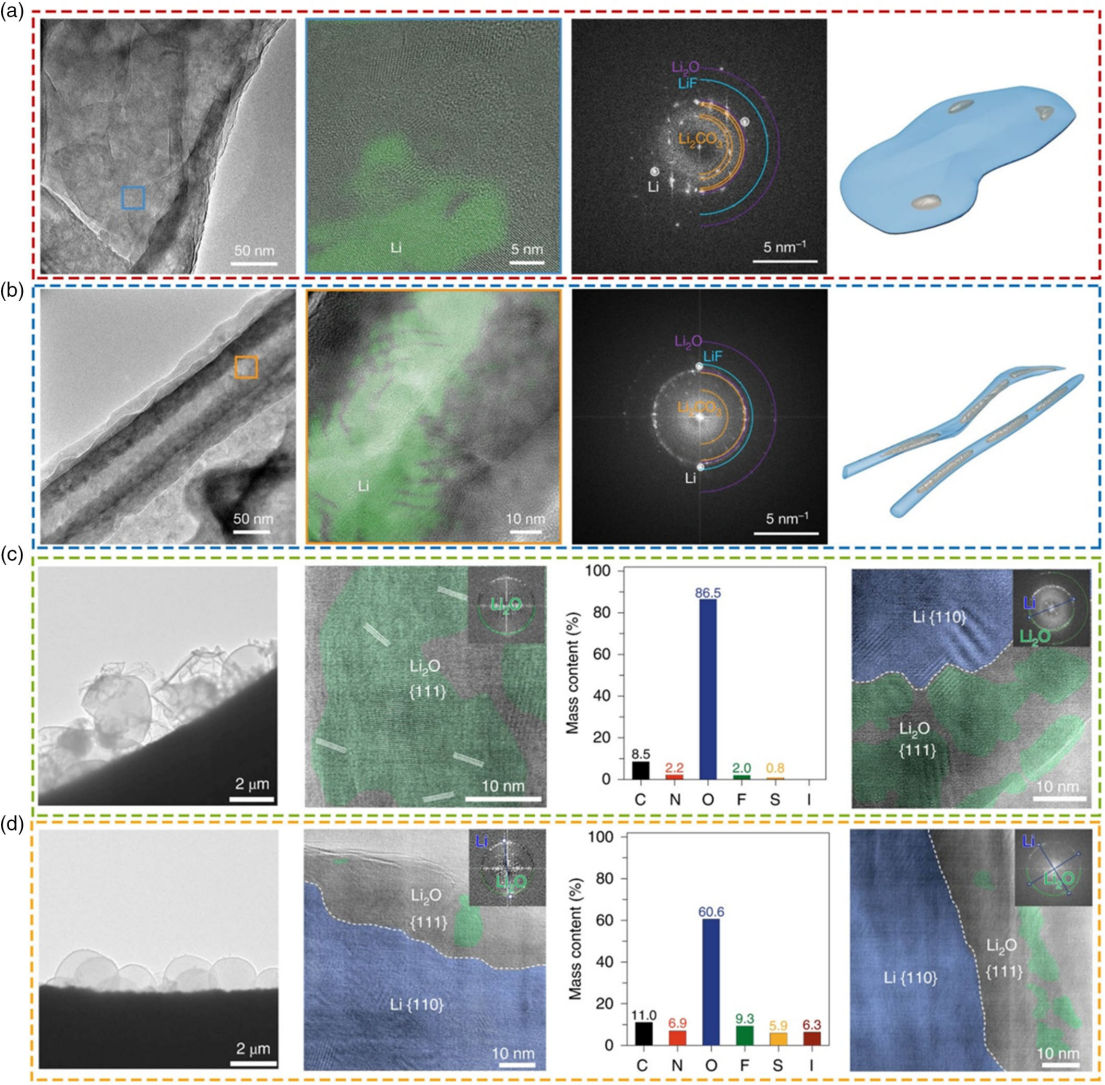
a,b) Cryo‐TEM images and schematic of inactive Li nanostructure generated in a) high‐concentration electrolyte and b) commercial carbonate electrolyte. Reproduced with permission.^[^ ^50^ ^]^ Copyright 2019, Springer Nature. c) Microstructures and components of different SEIs formed in ether (LiTFSI—DOL—DME) and carbonate (LiPF_6_—EC—EMC—DEC) electrolytes. d) Microstructures and components of different SEIs formed in iodine‐containing ether and carbonate electrolytes. c,d) Reproduced with permission.^[^ ^57^ ^]^ Copyright 2021, Springer Nature.

Although the generation and accumulation of inactive Li is a key factor in Li anode failure,^[^ 54, 55, 56 ^]^ the knowledge about it is very limited. Based on cryo‐EM observations, Meng and coworkers identify the unreacted metallic Li^0^ as the predominant source of inactive Li and capacity loss.^[^ ^50^ ^]^ It shows that the lamellar dead Li^0^ presents in high‐concentration electrolyte (**Figure** **3**a), while the dead Li^0^ in commercial carbonate electrolyte remains the whisker‐like shape (Figure 3b). Compared with the dead Li^0^ in carbonate electrolyte, there is only a much smaller area of dead Li^0^ in high‐concentration electrolyte, indicating that most of the deposited Li is successfully stripped and thus corresponding to a higher CE. The fast Fourier transform pattern and HRTEM images demonstrate that these SEIs are mainly composed of Li_2_CO_3_ and Li_2_O, which also include LiF and other amorphous organics species. By contrast, the Li deposits with whisker morphology and large tortuosity tend to generate more unreacted dead Li^0^ in the stripping process. Thus, they propose that an ideal deposited Li framework will promote structural connectivity and slow down the formation of inactive Li, especially the unreacted dead Li^0^. The Li deposits should maintain a columnar structure with large particle size and minimum curvature, which can reduce the presence of dead Li^0^. In addition, the SEI should be chemically and spatially homogeneous so that Li ions can be uniformly stripped. The SEI should also be mechanically elastic enough to accommodate volume change because the Li at the tip of column or whiskers could fail to keep up with the rapid stripping process, resulting in dead Li^0^ formation and low CE. The correlation between structure and performance provides an important reference for the design and optimization of battery materials and the development of safe and efficient LMBs.

Recently, Tao and coworkers quantify the internal components of the SEI layer by means of cryo‐TEM and determine their relationship with the formation of electrically isolated inactive Li.^[^ ^57^ ^]^ Cryo‐TEM shows that the outermost SEI will collapse once the Li deposits were stripped in the ether electrolyte (Figure 3c). In subsequent Li deposition, there will form a new SEI and the previous collapsed SEI becomes the so‐called dead SEI due to its inability to protect the active Li. HRTEM images reveal that these SEIs formed in LiTFSI—DOL—DME and carbonate electrolytes are all dominated by Li_2_O nanocrystallines. The cryo‐STEM EDS result clarifies that there is a high content of oxygen within the Li deposits in ether electrolyte. In addition, the electrochemically insulated dead Li^0^ within dead SEI will eventually evolve into dead Li^0^. Thus, they demonstrated an effective strategy of rejuvenating the dead Li from dead SEI and dead Li^0^ by iodine redox chemistry (Figure 3d). The introduction of iodine allows neat Li microspheres grew on the Cu grid without dead SEI and dead Li^0^. Meanwhile, the content of Li_2_O nanocrystallines within the SEI decreases significantly in both LiTFSI—DOL—DME and carbonate electrolytes and the relevant O/C mass ratio in the deposited Li is markedly reduced as well. Finally, owing to this design, inactive Li is spontaneously transferred to the high‐voltage cathode and then revitalized to compensate for the Li loss, leading to remarkably improved cyclability of LMBs.

## 4 The Identification of SEI

### 4.1 Electrolyte Engineered SEI

**Figure 4:**
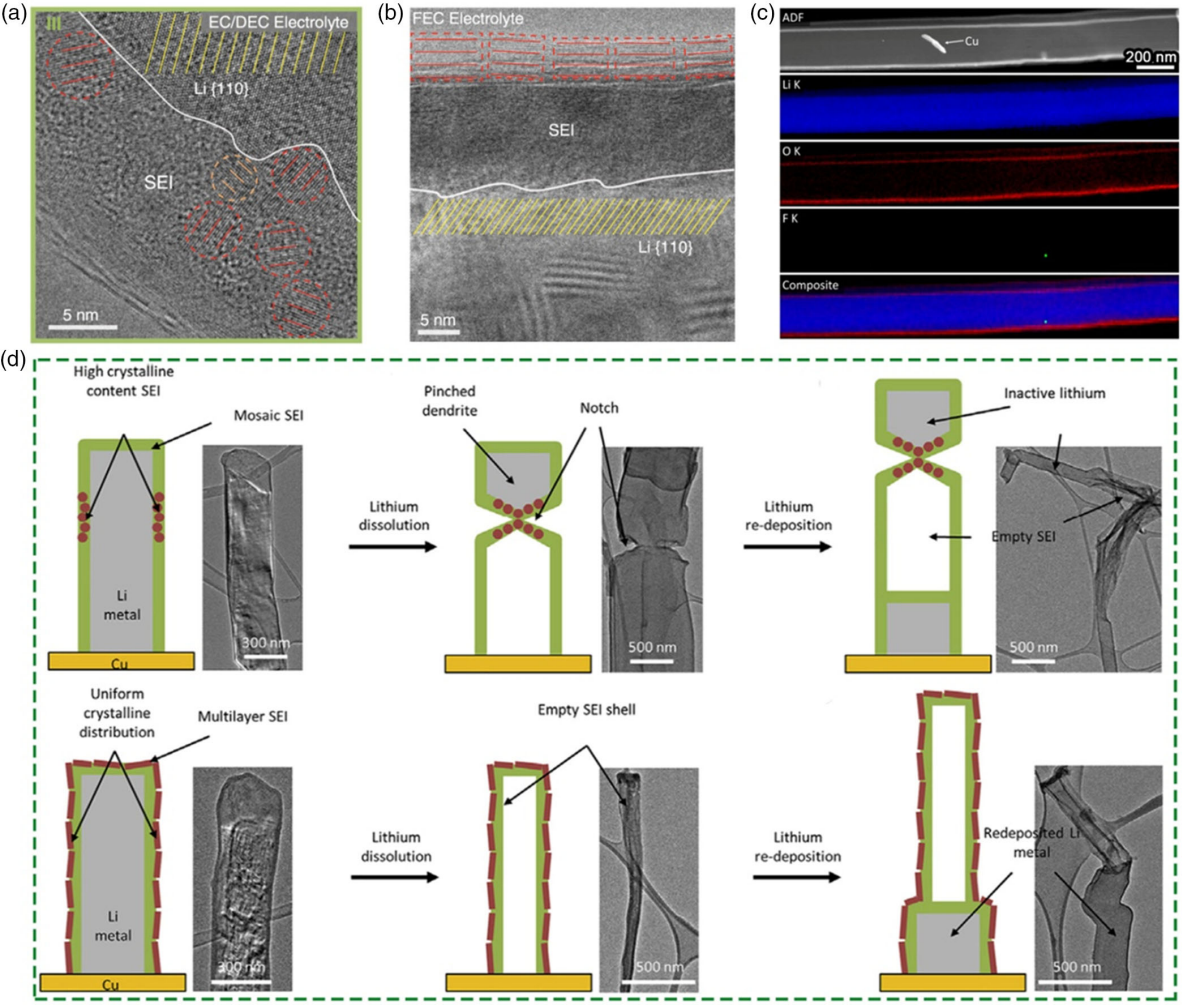
a,b) Cryo‐TEM images of the SEI formed in a) carbonate electrolyte and b) the electrolyte with FEC additives. a,b) Reproduced with permission.^[^ ^31^ ^]^ Copyright 2017, AAAS. c) Cryo‐STEM EELS mapping of the Li metal SEI in carbonate electrolyte with FEC. Reproduced with permission.^[^ ^63^ ^]^ Copyright 2020, American Chemical Society. d) Cryo‐TEM image and schematic of nonuniform and uniform Li stripping and plating through mosaic and multilayer SEI, respectively. Reproduced with permission.^[^ ^53^ ^]^ Copyright 2018, Elsevier.

The physicochemical properties of the SEI layer are largely determined by the electrolyte composition. And electrolyte additives have long been considered as a conventional strategy for improving the structural composition of the SEI and thus achieving stable Li metal anode.^[^ 58, 59, 60, 61 ^]^ In the beginning, Cui and coworkers observe the SEI nanostructure in the carbonate electrolyte with or without FEC additive. In EC—DEC electrolyte, it shows that there possess a typical mosaic structure with inorganic components (Li_2_O and Li_2_CO_3_) randomly distributed in the organic matrix (**Figure** **4**a). A more ordered multilayer SEI structure is formed in the FEC‐modified electrolyte, consisting of an amorphous polymer inner layer and an inorganic layered Li_2_O outer layer (Figure 4b). Nevertheless, the LiF lattice cannot be detected although it is considered to be the main reason for improved battery performance.^[^ ^62^ ^]^

To figure out the location of LiF, Cui et al. further investigate the dense SEI through cryo‐STEM and EELS.^[^ ^63^ ^]^ The result shows that highly localized F signals are found on the Li surface in some regions, indicating a sparse and local precipitation of LiF on the active material surface (Figure 4c). To this end, they define this larger length scale as the indirect SEI regime. The authors suggest that LiF is not the main contribution of anode passivation, nor does it affect the Li^+^ transport across the dense SEI film. These findings provide a new perspective for multiscale analysis of SEI structures and may change the traditional understanding of composition distribution in SEI and its effect on anode stability. In addition, they also proposed a solubility‐mediated sustained‐release method in which nitrate additives were stably dissolved in carbonate electrolyte to stabilize the Li metal anode.^[^ ^64^ ^]^ Cryo‐TEM results show that this method can change the nucleation of Li from dendritic to spherical. The out layer of the SEI consists of highly ordered crystalline Li_2_O, while the inner layer is amorphous matrix dispersed with Li_2_O and Li_2_CO_3_ nanocrystallites.

The decay and failure of the battery largely depend on the passivation layer formed on the electrode surface.^[^ 65, 66, 67 ^]^ Based on cryo‐TEM technique, Cui et al. further confirmed the morphological changes in two different SEI nanostructures formed with or without FEC additives during the stripping process, and correlated their remarkable effects with the performance of LMBs.^[^ ^53^ ^]^ They found that the Li stripping within the mosaic SEI in EC/DEC electrolyte is nonuniform (Figure 4d), resulting in the formation of inactive Li and the reduction of battery efficiency. In contrast, uniform Li stripping was observed for multilayer SEI in FEC‐modified electrolyte and the corresponding CE is also higher. In addition, by analyzing the distribution of inorganic nanoparticles (e.g., Li_2_O, Li_2_CO_3_) in SEI, they suggest that the density of inorganics in mosaic SEI is uneven and regions with high content of inorganic will cause fast Li‐ion transport. When the Li metal is stripped, the empty SEI shell collapses along with the formation of inactive Li. In the multilayer SEI, the density of inorganics is relatively uniform and the homogeneous Li stripping can be realized with less dead Li generation. This study explains the battery performance from the perspective of SEI nanostructure, and provides a new insight for the design of Li metal anode and electrolyte selection in the future.

**Figure 5:**
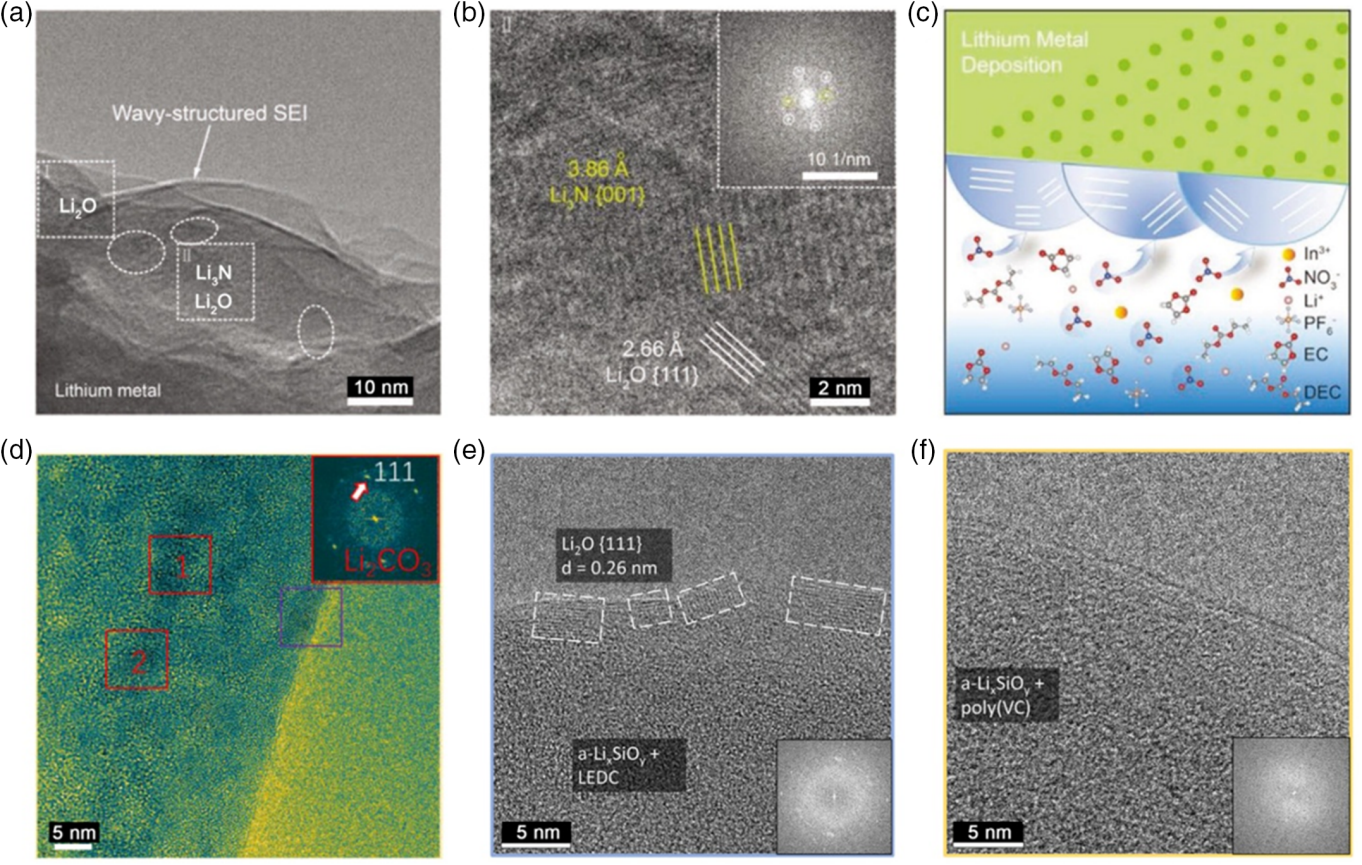
a) Cryo‐TEM image of the wavy‐structured SEI in SCCE electrolyte. b) Magnified image from (a). c) Schematic of the generated wavy SEI. a‐c) Reproduced with permission.^[^ ^68^ ^]^ Copyright 2020, Wiley‐VCH. d) HRTEM image revealing the interface between Li and the SEI in VC‐containing electrolyte. Reproduced with permission.^[^ ^71^ ^]^ Copyright 2019, American Chemical Society. e) Cryo‐TEM image of lithiated Si nanowires in EC/DEC electrolyte. f) Cryo‐TEM image of lithiated Si nanowires in EC/DEC electrolyte with FEC additives. e,f) Reproduced with permission.^[^ ^72^ ^]^ Copyright 2019, Elsevier.

In addition, Lu and coworkers introduced solubilizer indium trifluoromethanesulfonate (In(OTf)_3_) into conventional carbonate electrolyte (SCCE) to overcome the solubility barrier of LiNO_3_ cluster and achieved grain coarsening behavior of Li metal deposition.^[^ ^68^ ^]^ HRTEM results showed that SCCE electrolyte could promote the formation of nanowave‐structured SEI decorated with inorganic nanocrystallites of Li_2_O and Li_3_N (**Figure** **5**a,b). They suggested that the solvated In^3+^—NO_3_^−^ ionic complex in SCCE electrolyte could availably change the reaction sites in the solvation sheath. And NO_3_^−^ might undergo a site‐selective reactive at the inner Helmholtz plane, thus developing the inorganic SEI rich in nitrogen and oxygen (Figure 5c). This SEI was believed to promote the rapid transport of Li ions through the grain boundaries between multiple nanocrystalline components. Consequently, the wavy SEI with fast‐ion transport kinetics could achieve grain coarsening of Li deposition and complete dissolution of active Li metal upon cycling, which effectively alleviated the accumulation of “dead Li” and electrode pulverization in the LMBs. Similarly, they found that the introduction of solubilizer (including, tin trifluoromethanesulfonate (Sn(OTf)_2_)^[^ ^69^ ^]^ and tris(pentafluorophenyl)borane (TPFPB))^[^ ^70^ ^]^ to promote the solubility of LiNO_3_ additive significantly stabilized the SEI on the surface of Li metal anode, resulting in the uniform and dendrite‐free Li deposition. Furthermore, Wang et al. discovered that there were great differences in the SEI structures formed in VC‐free and VC‐containing electrolytes. It showed that the deposited Li metal in VC‐containing electrolyte was slightly oxidized with a nanoscale‐mosaic structure SEI (Figure 5d), which contributed to its high interfacial impedance. However, the Li deposits formed in VC‐free electrolyte was fully oxidized with multilayer nanostructured SEI.^[^ ^71^ ^]^

Except for the Li anode, Cui and coworkers reveal the dynamic SEI nanostructure of the Si anode in FEC‐modified electrolyte by cryo‐TEM.^[^ ^72^ ^]^ In typical EC/DEC electrolyte, the SEI on the lithiated Si surface is bilayer structure, composed of amorphous Li_*x*_SiO_*y*_ inner layer and an outer layer with amorphous Li ethylene dicarbonate and crystallized Li_2_O (Figure 5e). Although the SEI is composed of Li_2_O layer in the lithiated state, the Li_2_O layer derived from EC reduction is reversible. On delithiation, Li_2_O can react with Si to form Li_*x*_SiO_*y*_, and EC reduction products in the SEI will also be oxidized. Finally, the structure and chemical composition of SEI change significantly after delithiation, thus leading to poor cycling stability of Si anode. With the addition of FEC in electrolyte, there forms an amorphous SEI with organic polymerized vinylene carbonate (poly(VC)) deposited on the inner inorganic layer of Li_*x*_SiO_*y*_ (Figure 5f). During delithiation, the components within poly(VC) are stable against oxidation, which greatly prolongs the cyclic stability of the Si anode. Beyond that, Gu et al. investigate the effect of FEC additive on the SEI nanostructure of sodium (Na) metal electrode.^[^ ^73^ ^]^ It shows that there will develop a multilayer SEI with an outer NaF‐rich amorphous phase and an inner Na_3_PO_4_ phase in FEC‐modified electrolyte, which effectively prevents side reactions between Na metal and electrolyte.

**Figure 6:**
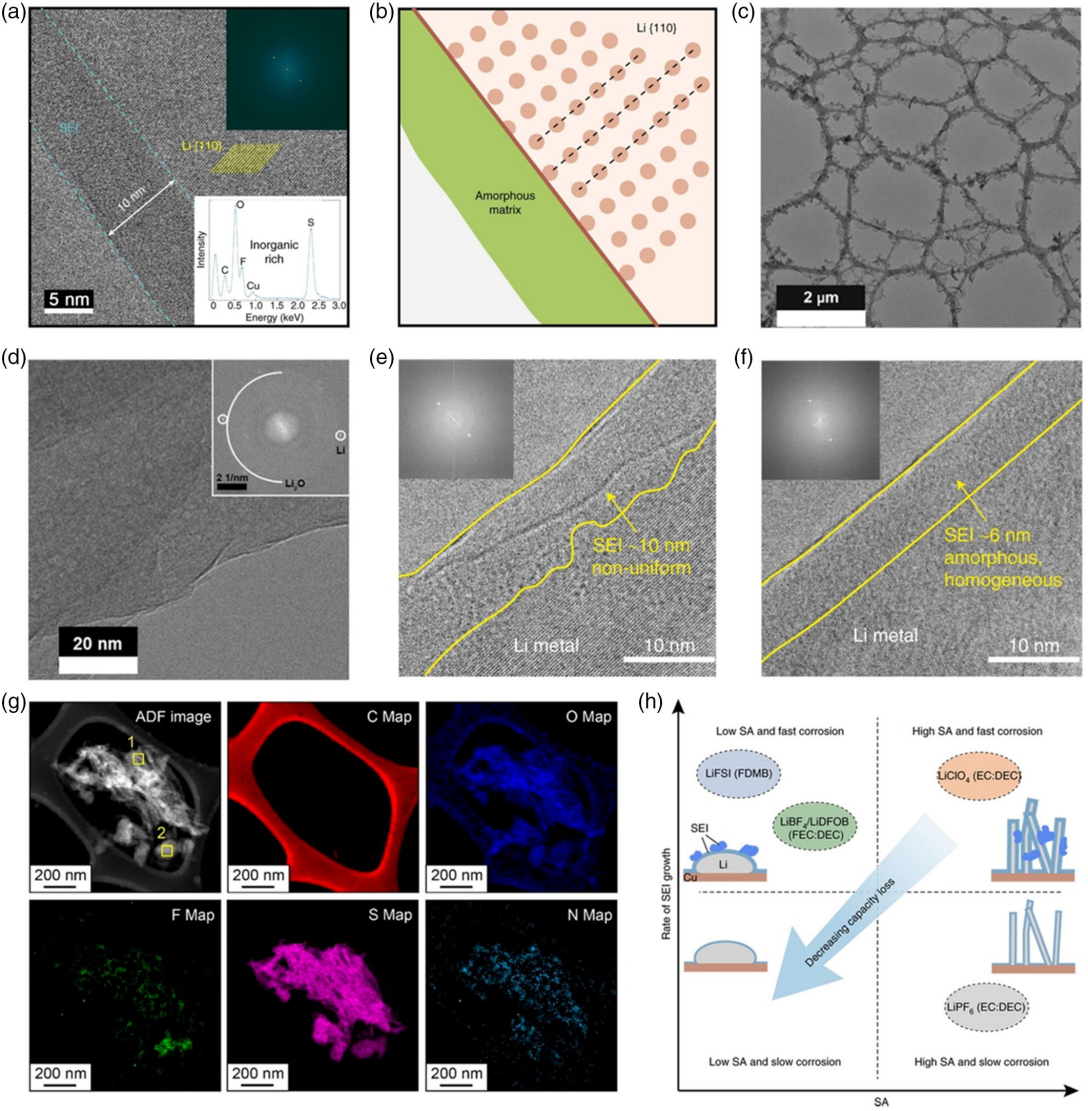
a) Cryo‐TEM image of the SEI layer in LiFSI/DME‐TFEO electrolyte. b) Schematic of the single‐layer SEI structure from (a). a,b) Reproduced with permission.^[^ ^77^ ^]^ Copyright 2019, Springer Nature. c,d) Cryo‐TEM images of c) Li deposits and d) the SEI formed in bisalt ether electrolyte. Reproduced with permission.^[^ ^78^ ^]^ Copyright 2019, Royal Society of Chemistry. e,f) Cryo‐TEM images of the SEIs formed in e) LiFSI/DME and f) LiFSI/FDMB electrolyte. Reproduced with permission.^[^ ^79^ ^]^ Copyright 2020, Springer Nature. g) Cryo‐STEM EELS mapping of the rSEI in the LiFSI/FDMB electrolyte. Reproduced with permission.^[^ ^80^ ^]^ Copyright 2021, American Chemical Society. h) Schematic of the relationship between the rate of SEI growth, surface area (SA) of Li and capacity loss of Li metal anodes in liquid electrolytes. Reproduced with permission.^[^ ^81^ ^]^ Copyright 2021, Springer Nature.

Apart from the additives, adjusting the concentration and composition of electrolyte is also the key to improve the cycling stability of LMBs.^[^ 74, 75, 76 ^]^ Some recent studies have shown that the formation of an amorphous SEI structure can be more conducive to stabilizing the Li metal anode through electrolyte regulation. Zhang et al. formed monolithic SEI membranes with an amorphous structure by using fluorinated orthoformate electrolyte.^[^ ^77^ ^]^ The cryo‐EDS results revealed that this SEI with higher levels of O—, F—, and S–containing compounds and these inorganic compounds in SEI were not crystallized (**Figure** **6**a). The highly homogeneous monolithic SEI not only prevents the formation of Li dendrites, but also minimizes the Li loss and volume expansion (Figure 6b). In comparison, the inhomogeneity of structure and composition for mosaic or multilayer SEI film will induce the uneven Li deposition and fast Li and electrolyte depletion. Finally, this monolithic SEI layer with abundant inorganic species can endow high CE of Li metal anodes and long‐term cycling stability of LMBs.

In addition, Xu and coworkers investigated the Li nucleation growth behavior in bisalt ether electrolytes (BSEE).^[^ ^78^ ^]^ When Li is deposited in the carbonate‐based electrolyte, cryo‐TEM shows that there will generate lots of Li dendrites with crystal SEI structure containing LiOH and Li_2_O. On the contrary, the nucleation of Li in BSEE is more uniform with nanosheet morphology (Figure 6c). HRTEM image shows that the deposited Li presents in the <110> crystalline orientation and its SEI mainly consists of crystalline Li_2_O (Figure 6d). As the morphology and bulk density of Li plating are the key factors in determining the efficiency and lifespan of batteries, they also used cryogenic focused ion beam (cryo‐FIB) to analyze the cross‐sectional morphologies of plated Li. For the carbonate electrolyte, the deposited Li metal is a continuous, highly porous structure of Li dendrites. There are obvious gaps between Li deposits and Cu foil throughout the whole structure. In contrast, Li plated in BSEE has a significant increase in density and thickness. It exhibits more uniform nucleation and deposition, consistent with its excellent electrochemical properties.

In addition, Bao et al. designed fluorinated 1,4‐dimethoxylbutane (FDMB) as the electrolyte solvent, making it possible to develop anode‐free LMBs with single‐solvent single‐salt formations at standard concentrations.^[^ ^79^ ^]^ According to the results of cryo‐TEM and cryo‐EDS, there will form a nonuniform SEI layer with wrinkles in LiFSI/DME electrolyte (Figure 6e). On the contrary, the amorphous and ultrathin SEI (≈6 nm) can be realized in FDMB‐based electrolyte (Figure 6f), which is rich in F, S, and O. This uniform SEI can effectively reduce the Li consumption of SEI formation in each cycle, and thus improve the CE of the battery. Furthermore, Cui et al. studied the Li cycling performance in LiFSI—FDMB electrolyte under different pressure by using anode‐free pouch cells.^[^ ^80^ ^]^ Combined with cryo‐STEM—EELS analysis, they suggest that the residual solid electrolyte interface (rSEI) in LiFSI/FDMB electrolyte is mainly composed of anion‐derived species and accumulated LiF nanoparticles (Figure 6g). Li metal can grow through the porous rSEI layer from the Cu substrate to reach the outer surface, thus ensuring stable and dense Li deposition. By comparison, the rSEI in EC/DEC electrolyte consists of a polymer skeleton that prevents the penetration of Li and therefore can only be deposited underneath the rSEI. Increased pressure will promote uniform Li deposition and higher CE by mechanically compacting the Li metal layers.

Although the optimized electrolyte can improve the structural properties of SEI and battery performance, the chemical corrosion still exists in the LMBs. Recently, Cui et al. reveal the effect of calendar ageing on the rechargeable LMBs and quantify the irreversible capacity loss of Li in various electrolyte chemicals.^[^ ^81^ ^]^ Studies have shown that, regardless of the electrolyte chemistry, Li metal loses at least 2–3% of its capacity after only 24 h of ageing. Combined with cryo‐EM results, it indicates that the chemical corrosion of the Li and the continuous growth of SEI result in the capacity loss. Electrolytes with long lifespan, such as LiFSI/FDMB electrolyte, do not necessarily form more chemically corrosion‐resistant SEI. Therefore, functional electrolytes must minimize both the growth rate of SEI and the surface area of electrodeposited Li (Figure 6h).

### 4.2 Artificial SEI

The native SEIs formed by the parasitic reaction between Li metal and liquid electrolyte are heterogeneous and poorly ion‐conductive, which not only impede the ion transport, but also accelerate the formation of Li dendrites and dead Li. Therefore, to realize the stable operation of Li anode, building artificial SEI with ionic conductivity and excellent stability on Li anode is considered to be an efficient and practical strategy.^[^ 82, 83, 84 ^]^

**Figure 7:**
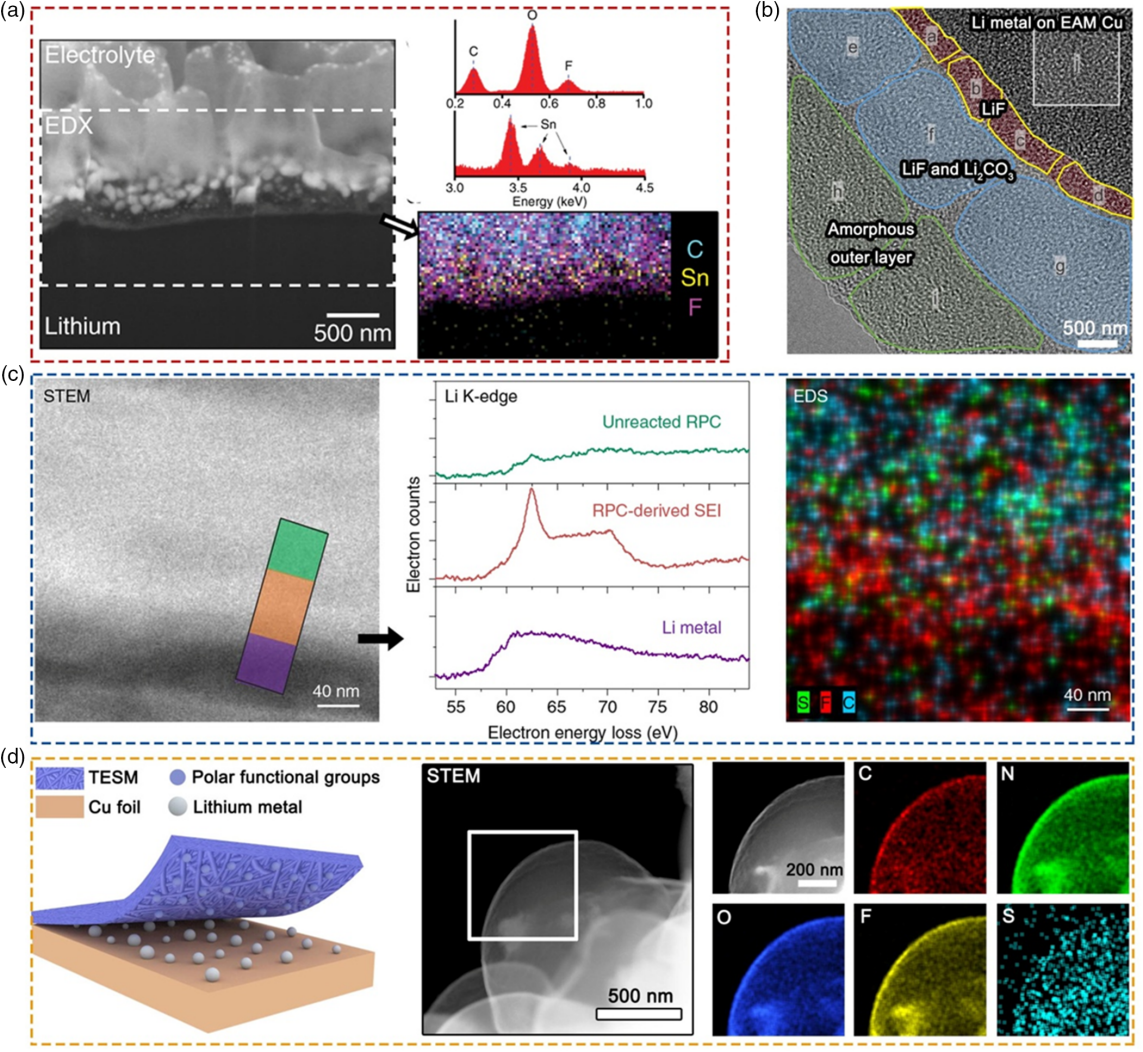
a) Cryo‐FIB‐SEM image and the relevant EDS spectra, elemental mapping of the tin‐protected Li. Reproduced with permission.^[^ ^85^ ^]^ Copyright 2018, Springer Nature. b) Cryo‐TEM image of the low‐temperature SEI formed on the EAM Cu. Reproduced with permission.^[^ ^90^ ^]^ Copyright 2020, Springer Nature. c) Interfacial chemical compositions of RPC‐derived SEI layer based on cryo‐STEM EELS and EDS. Reproduced with permission.^[^ ^91^ ^]^ Copyright 2019, Springer Nature. d) Schematic and cryo‐STEM of Li microspheres deposited in the presence of TESM, and the corresponding EDS elemental mapping. Reproduced under the terms of the CC‐BY 4.0 license.^[^ ^92^ ^]^ Copyright 2020, The Authors, published by Springer Nature.

First, Archer et al. reported an artificial interphase by a facile ion‐exchange chemistry and studied the cross section of Sn—Li anode by using cryo‐FIB‐scanning electron microscope (cryo‐FIB‐SEM).^[^ ^85^ ^]^ The interface morphology and element distribution characterized at cryogenic temperature indicated that there formed a trilayer structure (**Figure** **7**a). The top layer is a frozen electrolyte, and the bottom layer is Li metal. The middle is a Sn‐rich layer with a thickness of about 500 nm and is composed of nanoparticles (≈200 nm). EDS mapping confirms the presence of Sn in the nanoparticles, which are uniformly distributed on the Li surface. Eventually, this artificial SEI layer prevents the parasitic reactions and stabilizes the Li metal anode. At the same time, the results based on cryo‐FIB‐SEM combined EDS also demonstrated the uniform distribution of Al_2_O_3_,^[^ ^86^ ^]^ ionomer^[^ ^87^ ^]^ as artificial interface layer on the Li metal surface and NaBr coating^[^ ^88^ ^]^ on the Na anode surface, which can effectively reduce the dendritic deposition.

The low redox potential of Li metal makes it susceptible to corrosion. Cui and coworkers used cryo‐TEM to observe the evolution of Li deposits with the duration of rest in electrolyte, and proposed a Kirkendall‐type rapid Li corrosion mechanism.^[^ ^89^ ^]^ The results show that a galvanic process between Li and Cu substrate controls the corrosion within Li metal anode. They found that effective Li anticorrosion can be realized by introducing conformal LiF coating on Cu electrode. Therefore, to develop high‐performance LMBs, the effect of galvanic corrosion must be minimized in the battery. To this end, Wang and coworkers reported a method for stable cycling of LMBs at low temperatures by coating a monolayer of 1,3‐benzenedisulfonyl fluoride (EAM) on Cu current collector.^[^ ^90^ ^]^ With the assistance of EAM, cryo‐TEM shows that there will form a multilayered SEI, which possesses a LiF‐rich inner layer and amorphous outer layer embedded with Li_2_CO_3_ and LiF nanocrystals (Figure 7b). Consequently, the galvanic corrosion and self‐discharge of cells are suppressed and stable operation of Li metal anode can be maintained by EAM‐regulated interface.

In addition, Wang et al. reported a design by using reactive polymer composite (RPC) as SEI precursors.^[^ ^91^ ^]^ Cryo‐TEM image shows that the Li/RPC interface has three layers assigned to the unreacted RPC, RPC‐derived SEI, and inner Li layer, respectively. The RPC‐derived SEI about ≈90–120 nm thick is an amorphous layer embedded with LiF nanocrystals. Based on cryo–STEM EELS, they further studied the chemical composition of the RPC‐derived interface (Figure 7c). The Li K‐edge spectra taken from the boxed area show the peaks of Li metal, RPC‐derived SEI with LiF, and unreacted RPC, respectively. The three different layers also can be identified by its corresponding cryo‐STEM EDS image. Unlike the SEI formed by traditional electrolyte, the RPC‐derived SEI has excellent passivation performance, uniformity, and mechanical strength, and can realize stable Li metal anode even in poor electrolyte, limited Li, and high capacity.

In virtue of cryo–TEM, Tao and coworkers designed a biomacromolecule matrix obtained from the natural eggshell membrane to control Li growth and monitored the growth characteristics and orientation structure of Li crystals at the atomic scale (Figure 7d).^[^ ^92^ ^]^ It was found that the dendrite growth orientation, especially along the <111> crystal direction, was greatly inhibited under the induction of trifluoroethanol‐modified eggshell membrane (TESM). In addition, the mass ratio of N to C elements in the SEI films formed without or with TESM was compared by monitoring the composition changes in the SEI at different potentials. They found that the mass ratio of N to C in SEI assisted by TESM at −0.05 V increased significantly, suggesting that the TESM species participates in SEI. TESM macromolecules embedded in the SEI may help to provide a uniform flux of Li ions, leading to the spherical Li morphology. In addition, they constructed a fast‐ion conducting interface by employing aluminum silicate (ASO) fibers to address the inherent issue of Li dendrite growth.^[^ ^93^ ^]^ The cryo‐TEM results show that the ASO‐regulated SEI had an inorganic‐rich structure dispersed with amorphous lithiated ASO species, Li_2_O and Li_2_CO_3_ nanocrystalline. On this basis, they also investigated the effects of various interface layers on the SEI nanostructure.^[^ 94, 95 ^]^ They found that the soluble components in these interfacial layers were involved in the formation of SEI during the electrochemical cycling, which effectively promoted the rapid transport of Li ions and better performance.

### 4.3 Host Regulated SEI

As the metallic Li is host‐less, serious volume changes will occur during the plating/stripping process, thus leading to the repeatedly breakage‐repair process of SEI and battery failure.^[^ 96, 97, 98 ^]^ Host with relatively high surface area and lithiophilic property has been shown to be effective in reducing the local current density and homogenizing the ionic flux.^[^ 99, 100 ^]^ Subsequently, it can markedly prolong the Sand's time of dendrite growth and inhibit the formation of Li dendrites. Thereupon, constructing a stable host for Li metal anodes can be favorable strategy for enhancing the SEI layer and cycling performance of battery.^[^ 101, 102, 103, 104 ^]^

**Figure 8:**
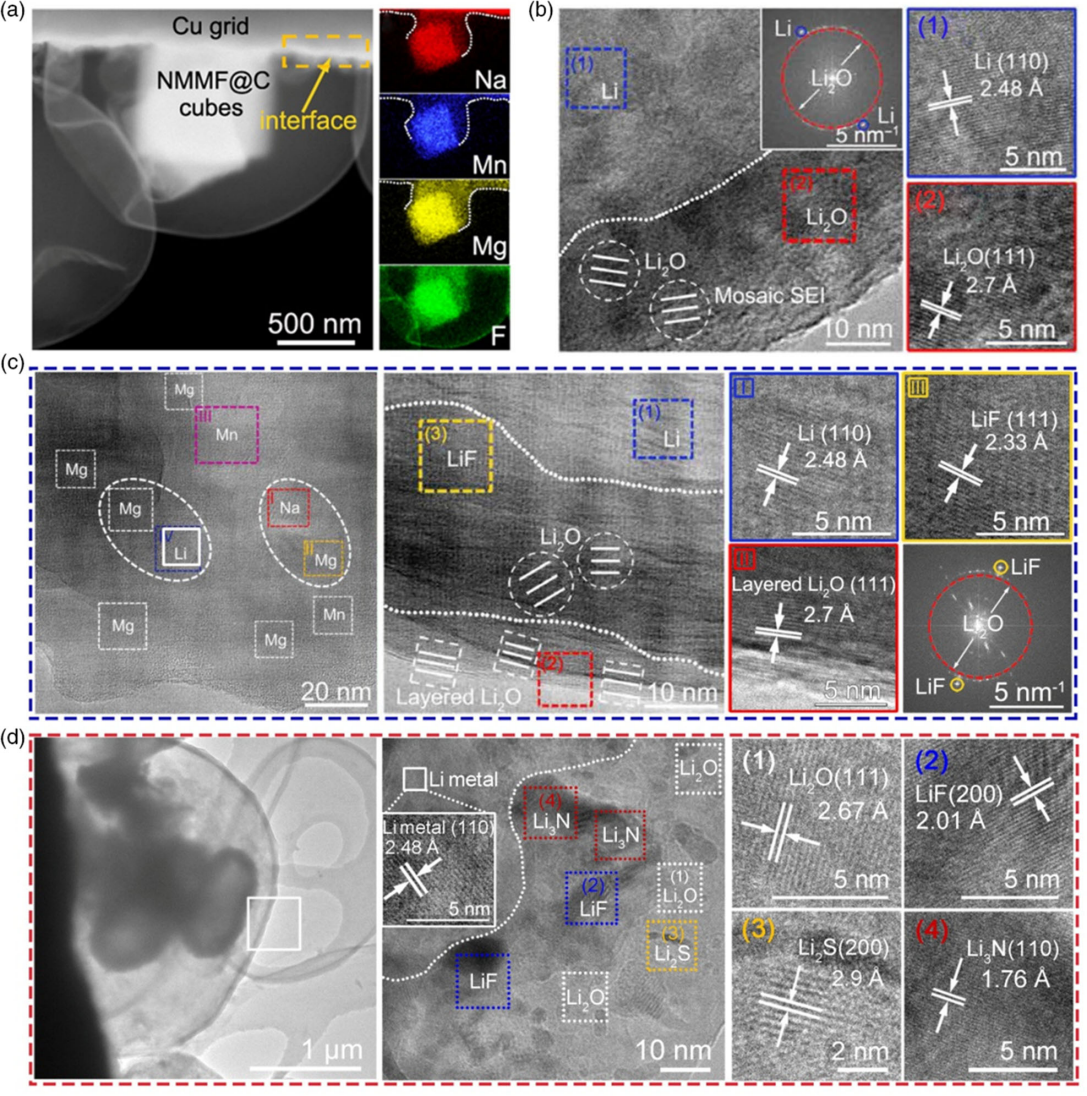
a) Cryo‐STEM image of Li deposites on the NMMF@C‐modified Cu electrode and the corresponding elemental mapping images. b) Cryo‐TEM showing the SEI formed on bare Cu grid. c) HRTEM images of the formed metal layer and LiF‐involved SEI layer regulated by NMMF@C. a‐c) Reproduced with permission.^[^ ^105^ ^]^ Copyright 2020, AAAS. d) Cryo‐TEM image of Li deposits on C@MoS_2_/S‐modified Cu grid and the relevant HRTEM image of SEI structure with various crystallines of Li_2_O, LiF, Li_2_S, and Li_3_N. Reproduced with permission.^[^ ^106^ ^]^ Copyright 2020, Wiley‐VCH.

Thus, to improve the stability of Li metal anode, Tao and coworkers designed a novel metal fluoride spansules (NaMg(Mn)F_3_@C, NMMF@C), which could enable persistent release of functional ions into the electrolyte.^[^ ^105^ ^]^ To understand the protective effects, the Li deposits in NMMF@C were observed by cryo‐STEM combined with EDS.^[^ ^105^ ^]^ As shown in **Figure** **8**a, dendrite‐free morphology was observed on the NMMF@C‐modified Cu grid and the deposited Li metal was spherical. The associated element mapping confirmed that the metal ions in NMMF@C could be released slowly. HRTEM image showed that the SEI generated on bare Cu grid was a typical mosaic structure with unevenly distributed inorganic (mainly Li_2_O) and organic components (Figure 8b). However, HRTEM observations confirmed that the released metal ions would in situ form a metal layer on the interface of NMMF@C and Cu grid (Figure 8c), which could guide the dendrite‐free Li growth behavior. In addition, there formed a unique LiF‐involved bilayer SEI structure on NMMF@C‐modified Cu grid, which would stabilize the Li/electrolyte interface and significantly improve the stability of the Li metal anodes.

Furthermore, they also designed a hollow nanostructure based on hierarchical MoS_2_ hollow carbon particles preloaded with sulfur (C@MoS_2_/S) for Li storage (Figure 8d).^[^ ^106^ ^]^ The observation of cryo‐TEM showed that the hollow C@MoS_2_ could be used as a good scaffold for repeated deposition of Li metal. In the initial nucleation stage, Li ions will intercalate into the MoS_2_ nanosheets, which may benefit to weaken the interfacial resistance and enable fast Li diffusion. In addition, the in situ formed Li_2_S with higher ionic conductivity and electrochemical stability will eliminate the preferred nucleation sites of Li dendrites. More importantly, the coated S will gradually release Li polysulfides during cycling and promote the formation of SEI layer embedded with crystalline hybrid Li‐based components, including LiF, Li_2_S, and Li_3_N. Compared with the mosaic SEI structure formed on bare Cu, the LiF—Li_2_S—Li_3_N‐riched SEI layer assisted by C@MoS_2_/S can realize the inhibition of Li dendrite growth and the stabilization of Li metal anode.

### 4.4 Temperature‐Dependent SEI

**Figure 9:**
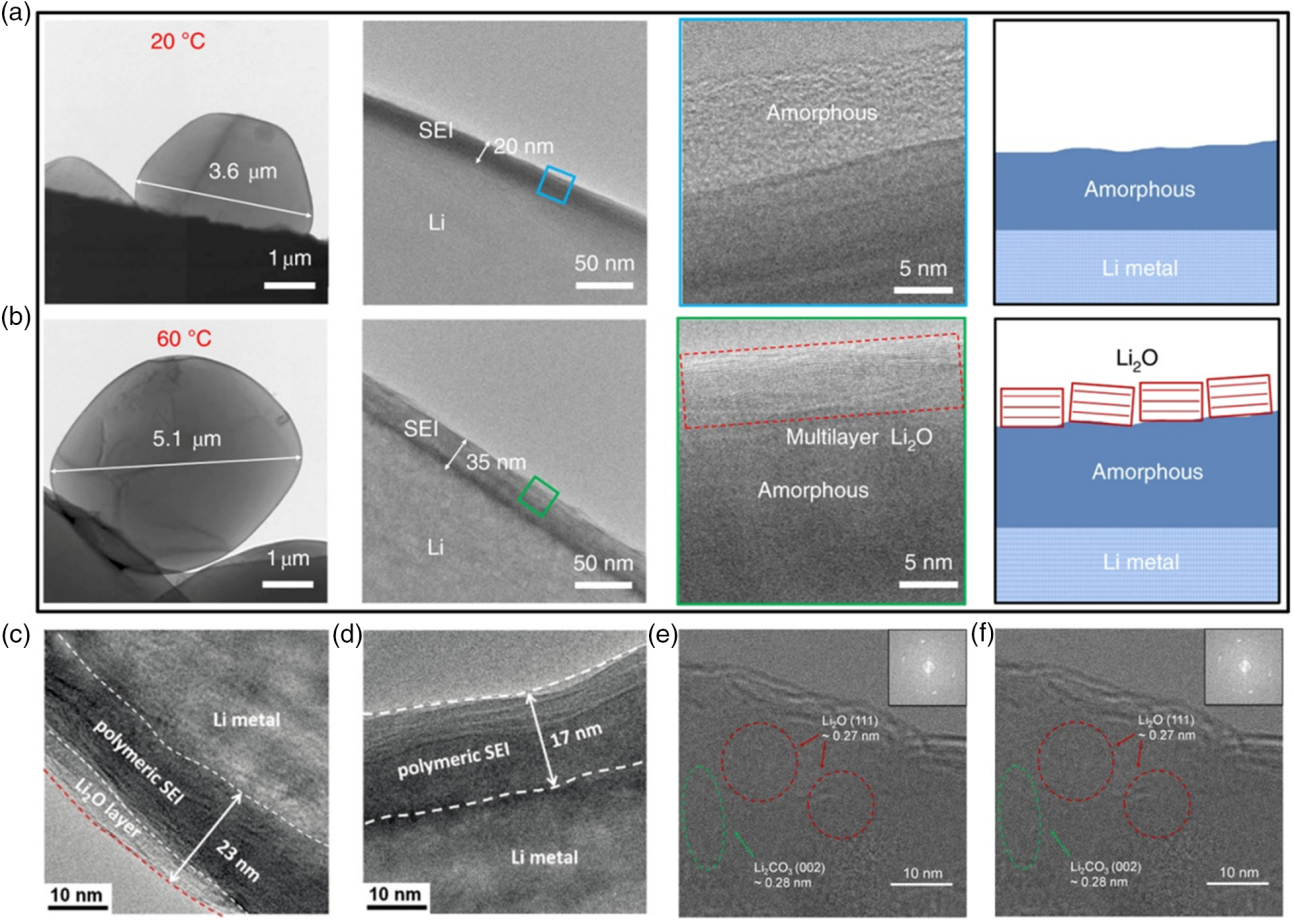
a,b) Cryo‐TEM images of Li deposits and the corresponding SEI nanostructure formed at a) 20 °C and b) 60 °C in ether‐based electrolyte. Reproduced with permission.^[^ ^109^ ^]^ Copyright 2019, Springer Nature. c,d) High‐resolution cryo‐TEM images of SEI formed at c) 60 °C and d) 20 °C in ether‐based electrolyte. Reproduced with permission.^[^ ^110^ ^]^ Copyright 2019, Wiley‐VCH. e,f) Cryo‐TEM images of SEI formed at e) 20 °C and f) −40 °C in DOL/DME electrolyte. Reproduced with permission.^[^ ^111^ ^]^ Copyright 2019, American Chemical Society.

The Li‐ion diffusion and interface reaction in the battery are always highly temperature‐dependent,^[^ 107, 108 ^]^ thus the operating temperature is the key factor that affects the property of SEI. It is generally believed that the cycling stability of the battery will decline with the increase in side reactions induced by the rising temperature. Cui and coworkers found that LMBs operating at high temperatures actually improved the cycling performance.^[^ ^109^ ^]^ The cryo‐TEM results showed that the size of Li particles deposited at 60 °C was larger than that Li deposits formed at 20 °C (**Figure** **9**a,b). The SEI layer formed at 20 °C was an amorphous polymeric interphase, while the formed SEI at 60 °C had a thicker nanostructure with an amorphous inner layer and layered Li_2_O outer layer. As a result, this favorable SEI at high temperatures can provide faster kinetics, inhibit successive side reactions, and guarantee excellent cycle stability.

In the meantime, Wang et al. also investigated the temperature‐dependent Li growth behavior and enabled a stable Li metal anode by raising operating temperature.^[^ ^110^ ^]^ Similarly, there will form an amorphous SEI at 20 °C and the SEI formed at 60 °C is made up of an amorphous polymeric interphase and a Li_2_O outer layer (Figure 9c,d). In addition, McDowell's team used cryo‐TEM to study SEI characteristics at low temperatures.^[^ ^111^ ^]^ It was found that the SEI generated at 20 and −40 °C showed mosaic pattern. However, Li_2_O and Li_2_CO_3_ crystals were observed in SEI formed at 20 °C, while LiF crystals were mainly observed in SEI formed at −40 °C (Figure 9e,f). They believed that the differences observed in SEI chemistry and structure could be ascribed to changes in the kinetics involved in SEI formation at different temperatures. Further observation revealed that the SEI formed in FEC‐modified electrolyte at −40 °C mainly consisted of LiF and Li_2_CO_3_ crystals embedded in amorphous matrix, primarily because of the modified solvation behavior and SEI formation process.^[^ ^112^ ^]^

## 5 Nanostructure Determination of CEI

**Figure 10:**
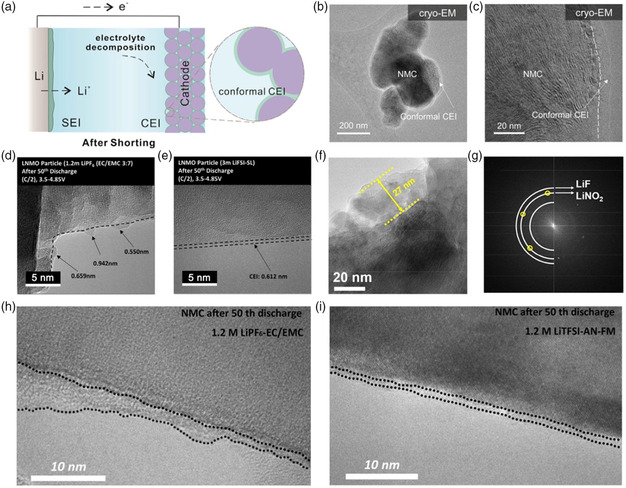
a) Schematic diagram for the conformal CEI formed via electrochemical shorting. b,c) Cryo‐TEM images of conformal CEI formed on NMC electrodes. a‐c) Reproduced with permission.^[^ ^114^ ^]^ Copyright 2021, Elsevier. d,e) Cryo‐TEM images of CEI on the cycled LNMO particle in d) LiPF_6_/EC/EMC and e) LiFSI‐sulfolane electrolyte. Reproduced with permission.^[^ ^127^ ^]^ Copyright 2018, Elsevier. f,g) Cryo‐TEM image and corresponding SAED image of SPAN cathode covered with a CEI layer in Li—S batteries. Reproduced with permission.^[^ ^115^ ^]^ Copyright 2019, Elsevier. h,i) Cryo‐TEM images of the cycled NMC particle in h) carbonate‐based electrolyte and i) liquefied gas electrolyte. Reproduced with permission.^[^ ^116^ ^]^ Copyright 2020, Royal Society of Chemistry.

Similar to the SEI, CEI is formed on the cathode surface due to electrolyte oxidation at high operating voltages. The nanostructure and composition of CEI also determine the cycling performance of cathode.^[^ ^113^ ^]^ Recently, Cui's group used cryo‐EM to visualize the CEI in its original state.^[^ ^114^ ^]^ Under normal operating conditions, authors found that there was no dense coating on the cathode surface at the single‐particle level in the carbonate electrolyte. However, there will in situ form stable and conformal CEI after performing simple external short circuit (**Figure** **10**a). The CEI is approximately 5–10 nm thick and most of it is mostly amorphous (Figure 10b,c), which helps to improve overall capacity retention of the battery.

Xu et al. explored a carbonate‐free and sulfone‐based electrolyte system to enable the high‐voltage LiNi_0.5_Mn_1.5_O_4_ (LNMO) cathode. Cryo‐TEM images exhibited that the generated CEI in carbonate electrolyte presented significant changes in thickness and uniformity with partial exposure of the LNMO cathode to electrolyte (Figure 10d). On the contrary, the thickness of CEI formed in LiFSI‐sulfone electrolyte was more uniform and conformal (Figure 10e). Due to the high oxidation potential of sulfone complexed with Li^+^ and the polymerization of sulfone, this electrolyte design can realize the improved anodic stability and subsequently ensure excellent high‐voltage and high‐temperature stability in full cells.

In addition, Liu and coworkers combined cryo‐EM to show that the sulfide polyacrylonitrile (SPAN) cathode formed a crystalline CEI consisting of LiF and LiNO_2_ in a high concentration of ether‐based electrolyte (Figure 10f,g).^[^ ^115^ ^]^ This CEI can effectively prevent the formation of soluble polysulfides and make the Li/SPAN battery achieve stable electrochemical performance. Meng's group has developed a unique liquefied gas electrolyte that supports rapid ion migration and is compatible with both Li metal anodes and high‐voltage cathodes.^[^ ^116^ ^]^ The cycled cathode NMC622 was observed by cryo‐TEM. It was found that the thickness and distribution of CEI in the carbonate‐based electrolyte changed significantly after 50 cycles (Figure 10h). In contrast, the CEI produced in liquefied gas electrolyte was more uniform in thickness and had better surface coverage (Figure 10i). Therefore, optimizing electrolyte chemistry to form a benign CEI layer is a promising strategy to develop long‐term LMBs.

## 6 Interfacial Chemistry of Solid Electrolyte

Traditional liquid nonaqueous electrolyte possesses high volatility and low boiling point, which is one of the main causes for the ignition and even explosion of battery. All‐solid‐state LMBs are considered as a promising energy storage technology due to its high safety and high energy density.^[^ 117, 118, 119, 120 ^]^ As the core component of all‐solid‐state LMBs, the development of solid electrolyte has attracted great attention from scientific research and industry.^[^ 121, 122 ^]^ However, the characterization of solid electrolyte is still challenging to visualize their chemically sensitive interface under high energy electron beam.

**Figure 11:**
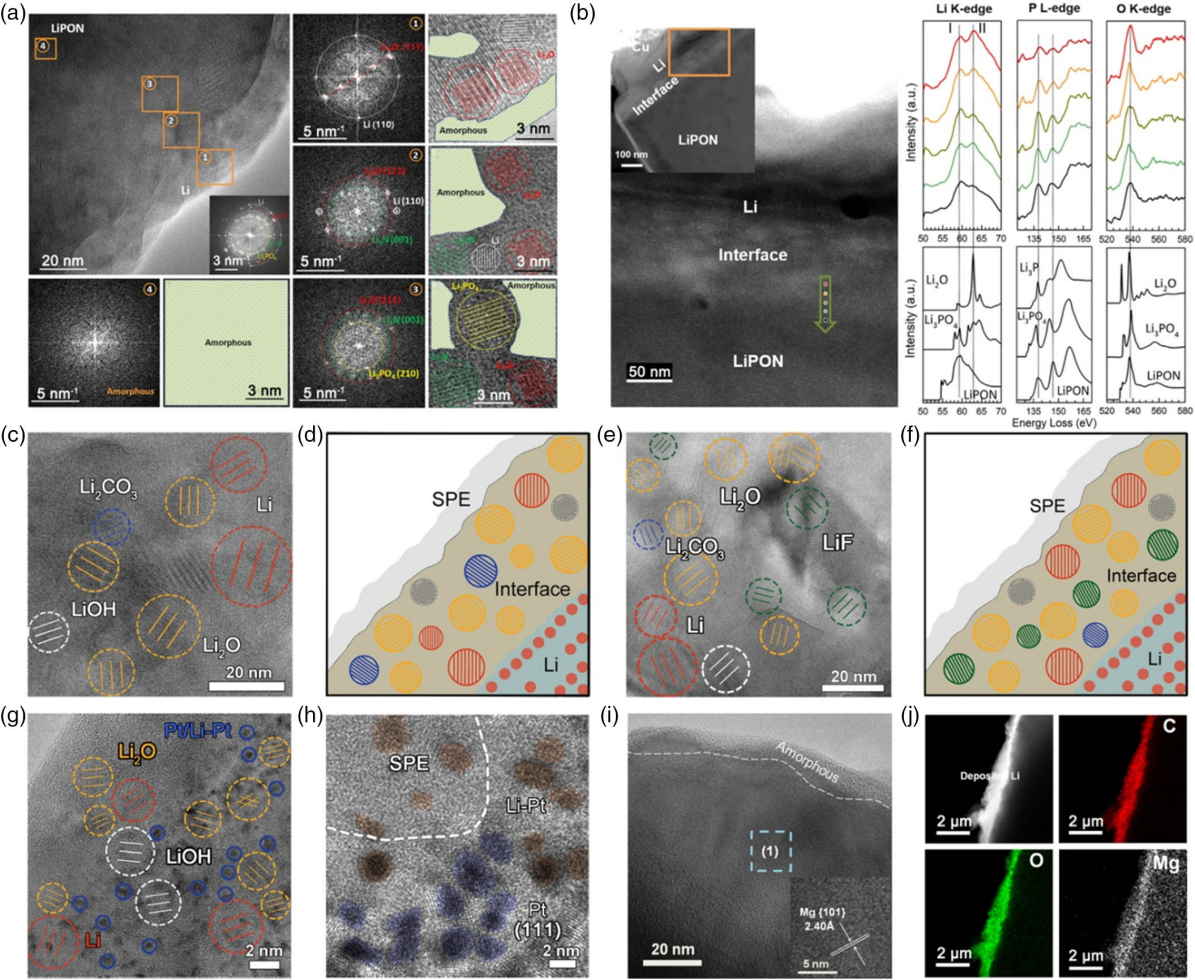
a) Cryo‐TEM image of the Li/LiPON interphase and the corresponding highlighted regions in (a). b) Cryo‐STEM—EELS analysis of the Li/LiPON interphase. a,b) Reproduced with permission.^[^ ^123^ ^]^ Copyright 2020, Elsevier. c) Cryo‐TEM image of Li/PEO interface in PEO—LiTFSI electrolyte and d) its schematic diagram. e) HRTEM image of Li/PEO interface in PEO—LiTFSI—Li_2_S electrolyte and f) its schematic diagram. c‐f) Reproduced with permission.^[^ ^124^ ^]^ Copyright 2020, Wiley‐VCH. g,h) HRTEM images of the interface between Li and Pt nanointerlayer‐modified SPE. Reproduced with permission.^[^ ^125^ ^]^ Copyright 2020, Elsevier. i) HRTEM images of the Li/PEO interface in PEO—LiTFSI—Mg(TFSI)_2_ electrolyte. j) The elemental mapping of Mg‐modified interface. i,j) Reproduced with permission.^[^ ^126^ ^]^ Copyright 2020, Elsevier.

Although solid‐state electrolyte LiPON can achieve good cycling performance of all‐solid‐state LMBs, how LiPON stabilizes Li metal and their interface chemistry with Li metal is still unclear. By means of cryo‐EM, Meng and coworkers characterized the composition evolution of the Li/LiPON interface and revealed a multilayer‐mosaic SEI structure (**Figure** **11**a).^[^ ^123^ ^]^ It can be seen that region 1 is mainly Li metal and Li_2_O, while Li_2_O, Li_3_N, and a small amount of Li metal are determined in region 2. Furthermore, Li_2_O, Li_3_N, and Li_3_PO_4_ are identified near LiPON region (region 3), and the amorphous structure of LiPON is detected in region 4. All nanocrystals are embedded in an amorphous matrix with a mosaic SEI distribution, which preserves the complete compactness of Li/LiPON interphase. In addition, they conduct the cryo‐STEM—EELS analysis to further understand the chemical evolution process of Li/LiPON phase. Figure 11b shows the EELS spectra of Li K‐edge, P L‐edge, and O K‐edge extracted from the point sampling in the interface region, which are consistent with the simulated EELS spectra. The peak I marked in the edge spectrum of Li K corresponds to the main peak in LiPON Li K‐edge spectrum, and peak II corresponds to the main peak of Li_2_O. The intensity ratio of peak I to peak II in the experimental spectra increases when moving from interface to LiPON region. This indicates that both Li_2_O and LiPON contribute to the experimental Li K‐edge spectra. And the contribution of Li_2_O decreases as it approaches the LiPON region. This result is consistent with the Cryo‐TEM results, where the interphase is confirmed to be nanocrystals distributed in amorphous LiPON matrix.

Inspired by the successful visualization of the interfacial structure of Li metal in liquid electrolyte, Tao and coworkers first disclose the interface of Li metal and solid electrolyte poly(ethylene oxide) (PEO) at atomic resolution through cryo‐TEM.^[^ ^124^ ^]^ Cryo‐TEM results indicate that there will form the typical mosaic model within the Li/PEO interface, which is composed of metallic Li and various inorganic nanocrystals (Li_2_O, LiOH, and Li_2_CO_3_) (Figure 11c,d). However, after artificially introducing Li_2_S additive, it shows that there will develop LiF‐enriched Li/PEO interface (Figure 11e,f), which can be attributed to the accelerated decomposition of TFSI^−^ assisted by Li_2_S. It is further confirmed that the produced LiF can restrain the cleavage of C—O bond in the polymer chain and avoid the continuous interface reaction between Li metal and PEO. Thus, the LiF‐enriched Li/PEO interface can effectively improve the performance of all‐solid‐state LMBs.

Meanwhile, to promote the interfacial stability, they build an artificial platinum (Pt) nanointerlayer between Li metal and PEO electrolyte.^[^ ^125^ ^]^ Finally, there will generate Li—Pt alloy nanoparticles within the Li/PEO interface (Figure 11g,h). Due to the favorable ion/electron conductive Li—Pt alloy, it is demonstrated that the modified Li/PEO interface present reduced interfacial impedance and side reactions, which can achieve dense Li deposition and higher capacity retention in all‐solid‐state batteries. They also introduced a Mg‐modified interface to guide uniform Li deposition by using Mg(TFSI)_2_‐modified PEO‐based electrolytes.^[^ ^126^ ^]^ Cryo‐TEM observations display that Mg dots were formed after the reduction of Mg^2+^ from Mg(TFSI)_2_ additive at the Li/PEO interface (Figure 11i). Cryo‐STEM EDS mapping shows that the uniform distribution of C, O, and Mg within the deposited Li further verifies the formation of the Mg‐modified interface (Figure 11j). This favorable interface consisting of crystalline Mg, Li_2_O, and Li dispersed into an amorphous polymer matrix will finally endow excellent cycling stability of all‐solid‐state LMBs.

## 7 Conclusions and Outlook

In summary, we have reviewed the application of advanced cryo‐EM techniques in the LMBs. The results show that the emergence of cryo‐EM provides a great convenience for revealing the underlying mechanism of fragile battery materials, which can preserve their native state without interference from air or electron beam. Based on this, we can realize the high‐resolution observation, such as the imaging of Li metal at atomic scale and the uncovering of mysterious interfacial nanostructures, which offers new thinking for the design and construction of electrode materials. We also can use cryo‐EM to solve many technical issues that have been difficult to achieve in the past, to make the correlation between battery performance and the microstructure and even the failure mechanisms.

Although numerous achievements have been made by using cryo‐EM, there are still many unknown challenges to be explored in battery systems. First of all, the application of cryo‐EM in the energy field is still in its infancy; optimizing imaging techniques to avoid unnecessary damage is the key to obtain objective information of materials. Meanwhile, cryo‐EM focused on local area of materials at micro‐ and nanoscale also has its limitation and sometimes the observations are unrepresentative. Furthermore, ex situ cryo‐EM characterization hampers our cognition on the dynamic electrochemical evolution of battery materials. Therefore, to solve these thorny issues, it is imperative for future technical innovation in cryo‐EM. 1) Technical upgrading. All the present researches are based on cryo‐TEM with a single‐tilt cryo‐transfer holder, which is obviously not conducive to the operability of the experiment. So, it is necessary to develop a double‐tilt holder to promote the high‐resolution imaging along zone axis. In addition, despite the cryo‐EM techniques allow for the atomic‐scale investigation, battery materials such as polymer electrolyte are still subject to the electron beam. Thus, the resolution of devices must be further improved to reduce the damage of thermal effect, so that we can explore more precise information about the lattice atomic arrangement, such as the defect and stress distribution. 2) Interworking of technologies. Although the combination of cryo‐TEM, cryo‐STEM, and cryo‐FIB can analyze the structural chemistry of the materials, it is necessary to combine conventional techniques, such as atomic force microscopy, nuclear magnetic resonance, Fourier transform infrared spectroscopy, and X‐ray photoelectron spectroscopy, to fully understand the properties of the battery materials. Through the multiangle characterization, we can understand the properties of materials more objectively and prevent the misleading results caused by the defects of the technology itself. 3) In situ devices. Existing cryo‐EM techniques are all measured under the stable state of the sample. To understand the electrochemical evolution of battery materials, it is necessary to develop in situ cryo‐EM, so as to realize the detection of samples with the cycle process, and understand the real‐time and dynamic interfacial chemistry. In addition, the in situ devices can also be used to learn various properties, such as mechanics, electricity, thermotics, and so on, thus forming a more comprehensive analysis system. However, it is still a great challenge in implementing in situ devices at low temperatures.

With the increasing demand of social energy, it is urgent to develop new energy storage equipment with high safety and high performance. Therefore, to achieve this goal, fundamental research that is still perplexing becomes particularly important. By summarizing the emerging applications of cryo‐EM in the field of energy, we hope that this work will point out possible directions for future research and accelerate the commercialization of high‐performance rechargeable batteries.

## Conflict of Interest

The authors declare no conflict of interest.

## Data Availability Statement

Data sharing is not applicable to this article as no datasets were generated or analyzed.

## References

[1] X.-B. Cheng , R. Zhang , C.-Z. Zhao , Q. Zhang , Chem. Rev. 2017, 117, 10403. 28753298 10.1021/acs.chemrev.7b00115

[2] J.-M. Tarascon , M. Armand , Nature 2011, 414, 359. 10.1038/35104644 11713543

[3] X. Zhang , Y. Yang , Z. Zhou , Chem. Soc. Rev. 2020, 49, 3040. 32292941 10.1039/c9cs00838a

[4] C. Jin , J. Nai , O. Sheng , H. Yuan , W. Zhang , X. Tao , X. W. Lou , Energy Environ. Sci. 2021, 14, 1326.

[5] S. Chang , M. Hou , B. Xu , F. Liang , X. Qiu , Y. Yao , T. Qu , W. Ma , B. Yang , Y. Dai , K. Chen , D. Xue , H. Zhao , X. Lin , F. Poon , Y. Lei , X. Sun , Adv. Funct. Mater. 2021, 31, 2011151.

[6] F. Wu , J. Maier , Y. Yu , Chem. Soc. Rev. 2020, 49, 1569. 32055806 10.1039/c7cs00863e

[7] G. Zheng , Y. Xiang , S. Chen , S. Ganapathy , T. W. Verhallen , M. Liu , G. Zhong , J. Zhu , X. Han , W. Wang , W. Zhao , M. Wagemaker , Y. Yang , Energy Storage Mater. 2019, 29, 377.

[8] B. Li , L. Kong , C. Zhao , Q. Jin , X. Chen , H. Peng , J. Qin , J. Chen , H. Yuan , Q. Zhang , J. Huang , InfoMat 2019, 1, 533.

[9] T. Liu , Q. Chu , C. Yan , S. Zhang , Z. Lin , J. Lu , Adv. Energy Mater. 2019, 9, 1802645.

[10] D. Lin , Y. Liu , Y. Cui , Nat. Nanotechnol. 2017, 12, 194. 28265117 10.1038/nnano.2017.16

[11] P. Zhai , L. Liu , X. Gu , T. Wang , Y. Gong , Adv. Energy Mater. 2020, 10, 2001257.

[12] Y. Hong , C. Zhao , Y. Xiao , R. Xu , J. Xu , J. Huang , Q. Zhang , X. Yu , H. Li , Batteries Supercaps 2019, 2, 638.

[13] D.-H. Liu , Z. Bai , M. Li , A. Yu , D. Luo , W. Liu , L. Yang , J. Lu , K. Amine , Z. Chen , Chem. Soc. Rev. 2020, 49, 5407. 32658219 10.1039/c9cs00636b

[14] W. Liu , W. Li , D. Zhuo , G. Zheng , Z. Lu , K. Liu , Y. Cui , ACS Cent. Sci. 2017, 3, 135. 28280780 10.1021/acscentsci.6b00389 PMC5324088

[15] J. Lu , T. Wu , K. Amine , Nat. Energy 2017, 2, 17011.

[16] Z. Li , Y. Zhang , T. Liu , X. Gao , S. Li , M. Ling , C. Liang , J. Zheng , Z. Lin , Adv. Energy Mater. 2020, 10, 1903110.

[17] H. Wu , H. Jia , C. Wang , J. Zhang , W. Xu , Adv. Energy Mater. 2020, 11, 2003092.

[18] Y. Jiang , B. Wang , P. Liu , B. Wang , Y. Zhou , D. Wang , H. Liu , S. Dou , Nano Energy 2020, 77, 105308.

[19] X. Guan , A. Wang , S. Liu , G. Li , F. Liang , Y.-W. Yang , X. Liu , J. Luo , Small, 2018, 14, 1801423. 10.1002/smll.201801423 30047235

[20] Q. Ma , Z. Chen , S. Zhong , J. Meng , F. Lai , Z. Li , C. Cheng , L. Zhang , T. Liu , Nano Energy 2021, 81, 105622.

[21] Z. Zeng , X. Liu , X. Jiang , Z. Liu , Z. Peng , X. Feng , W. Chen , D. Xia , X. Ai and H. Yang , Y. Cao , InfoMat 2020, 2, 984.

[22] Y. Guo , H. Li , T. Zhai , Adv. Mater. 2017, 29, 1700007. 10.1002/adma.201700007 28585291

[23] M. D. Tikekar , S. Choudhury , Z. Tu , L. A. Archer , Nat. Energy 2016, 1, 16114.

[24] J. Luo , E. Matios , H. Wang , X. Tao , W. Li , InfoMat 2020, 2, 1057.

[25] X. Wang , Y. Li , Y. S. Meng , Joule 2018, 2, 2225.

[26] H. Wang , X. Cao , H. Gu , Y. Liu , Y. Li , Z. Zhang , W. Huang , H. Wang , J. Wang , W. Xu , J.-G. Zhang Y. Cui , ACS Nano 2020, 14, 4601. 32271533 10.1021/acsnano.0c00184

[27] X. Ren , X. Zhang , R. Xu , J. Huang , Q. Zhang , Adv. Mater. 2020, 32, 1908293. 10.1002/adma.201908293 32249530

[28] C. Chen , M. Jiang , T. Zhou , L. Raijmakers , E. Vezhlev , B. Wu , T. U. Schülli , D. L. Danilov , Y. Wei , R.-A. Eichel , P. H. L. Notten , Adv. Energy Mater. 2021, 11, 2003939.

[29] D. Cressey , E. Callaway , Nature 2017, 550, 167. 29022937 10.1038/nature.2017.22738

[30] K. Murata , M. Wolf , Biochim. Biophys. Acta, 2018, 1862, 324-. 10.1016/j.bbagen.2017.07.020 28756276

[31] Y. Li , Y. Li , A. Pei , K. Yan , Y. Sun , C.-L. Wu , L.-M. Joubert , R. Chin , A. L. Koh , Y. Yu , J. Perrino , B. Butz , S. Chu , Y. Cui , Science 2017, 358, 506. 29074771 10.1126/science.aam6014

[32] Y. Liu , Z. Ju , B. Zhang , Y. Wang , J. Nai , T. Liu , X. Tao , Acc. Chem. Res. 2021, 54, 2088. 33856759 10.1021/acs.accounts.1c00120

[33] M. J. Zachman , Z. Tu , S. Choudhury , L. A. Archer , L. F. Kourkoutis , Nature 2018, 560, 345. 30111789 10.1038/s41586-018-0397-3

[34] Y. Xu , H. Wu , H. Jia , J.-G. Zhang , W. Xu , C. Wang , ACS Nano 2020, 14, 8766. 32598126 10.1021/acsnano.0c03344

[35] Y. Xu , H. Wu , H. Jia , M. H. Engelhard , J.-G. Zhang , W. Xu , C. Wang , Nano Energy 2020, 76, 105040.

[36] B. D. Levin , M. J. Zachman , J. G. Werner , S. Ritu , K. X. Nguyen , H. Yimo , X. Baoquan , M. Lin , A. Lynden , E. P. Giannelis , U. Wiesner , L. F. Kourkoutis , D. A. Muller , Microsc. Microanal. 2017, 23, 153. 10.1017/S1431927617000058 28228169

[37] X.-C. Liu , Y. Yang , J. Wu , M. Liu , S. P. Zhou , B. D. A. Levin , X.-D. Zhou , H. Cong , D. A. Muller , P. M. Ajayan , H. D. Abruña , F.-S. Ke , ACS Energy Lett. 2018, 3, 1325.

[38] N. Zhang , B. D. A. Levin , Y. Yang , D. A. Muller , H. D. Abruña , J. Electrochem. Soc. 2018, 165, A1656.

[39] J. Zheng , Z. Ju , B. Zhang , J. Nai , T. Liu , Y. Liu , Q. Xie , W. Zhang , Y. Wang , X. Tao , J. Mater. Chem. A 2021, 9, 10251.

[40] B. Han , Z. Zhang , Y. Zou , K. Xu , G. Xu , H. Wang , H. Meng , Y. Deng , J. Li , M. Gu , Adv. Mater. 2021, 33, 2100404. 10.1002/adma.202100404 33899278

[41] D. H. S. Tan , A. Banerjee , Z. Chen , Y. S. Meng , Nat. Nanotechnol. 2020, 15, 170. 32157239 10.1038/s41565-020-0657-x

[42] Y. Wu , N. Liu , Chem 2018, 4, 438.

[43] J. H. Um , S. Yu , Adv. Energy Mater. 2020, 10, 2070132.

[44] X. Chen , B. Zhao , C. Yan , Q. Zhang , Adv. Mater. 2021, 33, 2004128. 10.1002/adma.202004128 33432664

[45] G. Li , Adv. Energy Mater. 2021, 11, 2002891.

[46] J. Wen , Q. Zou , Y. Wei , J. Mech. Phys. Solids 2021, 153, 104481.

[47] W. S. LePage , Y. Chen , E. Kazyak , K.-H. Chen , A. J. Sanchez , A. Poli , E. M. Arruda , M. D. Thouless , N. P. Dasgupta , J. Electrochem. Soc. 2019, 166, A89.

[48] J. Z. Lee , T. A. Wynn , M. A. Schroeder , J. Alvarado , X. Wang , K. Xu , Y. S. Meng , ACS Energy Lett. 2019, 4, 489.

[49] Y. Li , W. Huang , Y. Li , W. Chiu , Y. Cui , ACS Nano 2020, 14, 9263. 32806083 10.1021/acsnano.0c05020

[50] C. Fang , J. Li , M. Zhang , Y. Zhang , F. Yang , J. Z. Lee , M.-H. Lee , J. Alvarado , M. A. Schroeder , Y. Yang , B. Lu , N. Williams , M. Ceja , L. Yang , M. Cai , J. Gu , K. Xu , X. Wang , Y. S. Meng , Nature 2019, 572, 511. 31435056 10.1038/s41586-019-1481-z

[51] D. Lu , Y. Shao , T. Lozano , W. D. Bennett , G. L. Graff , B. Polzin , J. Zhang , M. H. Engelhard , N. T. Saenz , W. A. Henderson , P. Bhattacharya , J. Liu , J. Xiao , Adv. Energy Mater. 2015, 5, 1400993.

[52] Y. Han , B. Liu , Z. Xiao , W. Zhang , X. Wang , G. Pan , Y. Xia , X. Xia , J. Tu , InfoMat 2021, 3, 155.

[53] Y. Li , W. Huang , Y. Li , A. Pei , D. T. Boyle , Y. Cui , Joule 2018, 2, 2167.

[54] J. Zhi , S. Li , M. Han , P. Chen , Sci. Adv. 2020, 6, eabb1342. 32821832 10.1126/sciadv.abb1342 PMC7413738

[55] C. Jin , O. Sheng , M. Chen , Z. Ju , G. Lu , T. Liu , J. Nai , Y. Liu , Y. Wang , X. Tao , Mater. Today Nano 2021, 13, 100103.

[56] Z. Yu , Y. Cui , Z. Bao , Cell Rep. Phys. Sci. 2020, 1, 100119.

[57] C. Jin , T. Liu , O. Sheng , M. Li , T. Liu , Y. Yuan , J. Nai , Z. Ju , W. Zhang , Y. Liu , Y. Wang , Z. Lin , J. Lu , X. Tao , Nat. Energy 2021, 6, 378.

[58] D. Luo , L. Zheng , Z. Zhang , M. Li , Z. Chen , R. Cui , Y. Shen , G. Li , R. Feng , S. Zhang , G. Jiang , L. Chen , A. Yu , X. Wang , Nat. Commun. 2021, 12, 186. 33420036 10.1038/s41467-020-20339-1 PMC7794354

[59] J. Wu , X. Li , Z. Rao , X. Xu , Z. Cheng , Y. Liao , L. Yuan , X. Xie , Z. Li , Y. Huang , Nano Energy 2020, 72, 104725.

[60] W. Chen , Y. Hu , W. Lv , T. Lei , X. Wang , Z. Li , M. Zhang , J. Huang , X. Du , Y. Yan , W. He , C. Liu , M. Liao , W. Zhang , J. Xiong , C. Yan , Nat. Commun. 2019, 10, 4973. 31672990 10.1038/s41467-019-12952-6 PMC6823444

[61] Z. Qian-Kui , Z. Xue-Qiang , Y. Hong , J.-Q. Huang , Small Sci. 2021, 10.1002/smsc.202100058.

[62] M. He , R. Guo , G. M. Hobold , H. Gao , B. M. Gallant , Proc. Natl. Acad. Sci. USA 2020, 117, 73. 31848237 10.1073/pnas.1911017116 PMC6955333

[63] W. Huang , H. Wang , D. T. Boyle , Y. Li , Y. Cui , ACS Energy Lett. 2020, 5, 1128.

[64] Y. Liu , D. Lin , Y. Li , G. Chen , A. Pei , O. Nix , Y. Li , Y. Cui , Nat. Commun. 2018, 9, 3656. 30194431 10.1038/s41467-018-06077-5 PMC6128910

[65] C. Zhao , Q. Zhao , X. Liu , J. Zheng , S. Stalin , Q. Zhang and L. A. Archer , Adv. Mater., 2020, 32, 1905629. 10.1002/adma.201905629 32053238

[66] G. Yasin , M. Arif , T. Mehtab , X. Lu , D. Yu , N. Muhammad , M. T. Nazir , H. Song , Energy Storage Mater. 2020, 25, 644.

[67] W. Xue , Z. Shi , M. Huang , S. Feng , C. Wang , F. Wang , J. Lopez , B. Qiao , G. Xu , W. Zhang , Y. Dong , R. Gao , Y. Shao-Horn , J. A. Johnson , J. Li , Energy Environ. Sci. 2020, 13, 212.

[68] W. Zhang , Z. Shen , S. Li , L. Fan , X. Wang , F. Chen , X. Zang , T. Wu , F. Ma , Y. Lu , Adv. Funct. Mater. 2020, 30, 2003800.

[69] W. Zhang , Q. Wu , J. Huang , L. Fan , Z. Shen , Y. He , Q. Feng , G. Zhu , Y. Lu , Adv. Mater. 2020, 32, 2001740. 10.1002/adma.202001740 32390225

[70] S. Li , W. Zhang , Q. Wu , L. Fan , X. Wang , X. Wang , Z. Shen , Y. He , Y. Lu , Angew. Chem. Int. Ed. 2020, 59, 14935. 10.1002/anie.202004853 32410377

[71] Y. Xu , H. Wu , Y. He , Q. Chen , J.-G. Zhang , W. Xu , C. Wang , Nano Lett. 2019, 20, 418. 31816244 10.1021/acs.nanolett.9b04111

[72] W. Huang , J. Wang , M. R. Braun , Z. Zhang , Y. Li , D. T. Boyle , P. C. McIntyre , Y. Cui , Matter 2019, 1, 1232.

[73] B. Han , Y. Zou , Z. Zhang , X. Yang , X. Shi , H. Meng , H. Wang , K. Xu , Y. Deng , M. Gu , Nat. Commun. 2021, 12, 3066. 34031418 10.1038/s41467-021-23368-6 PMC8144392

[74] Z. Wang , F. Qi , L. Yin , Y. Shi , C. Sun , B. An , H. Cheng , F. Li , Adv. Energy Mater. 2020, 10, 1903843.

[75] J. Qian , W. A. Henderson , W. Xu , P. Bhattacharya , M. Engelhard , O. Borodin , J.-G. Zhang , Nat. Commun. 2015, 6, 6362. 25698340 10.1038/ncomms7362 PMC4346622

[76] Z. Jiang , Z. Zeng , W. Hu , Z. Han , S. Cheng , J. Xie , Energy Storage Mater. 2021, 36, 333.

[77] X. Cao , X. Ren , L. Zou , M. H. Engelhard , W. Huang , H. Wang , B. E. Matthews , H. Lee , C. Niu , B. W. Arey , Y. Cui , C. Wang , J. Xiao , J. Liu , W. Xu , J.-G. Zhang , Nat. Energy 2019, 4, 796.

[78] J. Alvarado , M. A. Schroeder , T. P. Pollard , X. Wang , J. Z. Lee , M. Zhang , T. Wynn , M. Ding , O. Borodin , Y. S. Meng , K. Xu , Energy Environ. Sci. 2019, 12, 780.

[79] Z. Yu , H. Wang , X. Kong , W. Huang , Y. Tsao , D. G. Mackanic , K. Wang , X. Wang , W. Huang , S. Choudhury , Y. Zheng , C. V. Amanchukwu , S. T. Hung , Y. Ma , E. G. Lomeli , J. Qin , Y. Cui , Z. Bao , Nat. Energy 2020, 5, 526.

[80] H. Wang , W. Huang , Z. Yu , W. Huang , R. Xu , Z. Zhang , Z. Bao , Y. Cui , ACS Energy Lett. 2021, 6, 816.

[81] D. T. Boyle , W. Huang , H. Wang , Y. Li , H. Chen , Z. Yu , W. Zhang , Z. Bao , Y. Cui , Nat. Energy 2021, 6, 487.

[82] Y.-C. Yin , Q. Wang , J.-T. Yang , F. Li , G. Zhang , C.-H. Jiang , H.-S. Mo , J.-S. Yao , K.-H. Wang , F. Zhou , H.-X. JuYao , Nat. Commun. 2020, 11, 1761. 32273513 10.1038/s41467-020-15643-9 PMC7145840

[83] S. Liu , X. Ji , J. Yue , S. Hou , P. Wang , C. Cui , J. Chen , B. Shao , J. Li , F. Han , J. Tu , C. Wang , J. Am. Chem. Soc. 2020, 142, 2438. 31927894 10.1021/jacs.9b11750

[84] Z. Yu , D. G. Mackanic , W. Michaels , M. Lee , A. Pei , D. Feng , Q. Zhang , Y. Tsao , C. V. Amanchukwu , X. Yan , H. Wang , S. Chen , K. Liu , J. Kang , J. Qin , Y. Cui , Z. Bao , Joule 2019, 3, 2761.

[85] Z. Tu , S. Choudhury , M. J. Zachman , S. Wei , K. Zhang , L. F. Kourkoutis , L. A. Archer , Nat. Energy 2018, 3, 310.

[86] Z. Tu , M. J. Zachman , S. Choudhury , K. A. Khan , Q. Zhao , L. F. Kourkoutis , L. A. Archer , Chem. Mater. 2018, 30, 5655.

[87] Z. Tu , S. Choudhury , M. J. Zachman , S. Wei , K. Zhang , L. F. Kourkoutis , L. A. Archer , Joule 2017, 1, 394.

[88] S. Choudhury , S. Wei , Y. Ozhabes , D. Gunceler , M. J. Zachman , Z. Tu , J. H. Shin , P. Nath , A. Agrawal , L. F. Kourkoutis , T. A. Arias , L. A. Archer , Nat. Commun. 2017, 8, 898. 29026067 10.1038/s41467-017-00742-x PMC5638817

[89] D. Lin , Y. Liu , Y. Li , Y. Li , A. Pei , J. Xie , W. Huang , Y. Cui , Nat. Chem. 2019, 11, 382. 30664717 10.1038/s41557-018-0203-8

[90] Y. Gao , T. Rojas , K. Wang , S. Liu , D. Wang , T. Chen , H. Wang , A. T. Ngo , D. Wang , Nat. Energy 2020, 5, 534.

[91] Y. Gao , Z. Yan , J. L. Gray , X. He , D. Wang , T. Chen , Q. Huang , Y. C. Li , H. Wang , S. H. Kim , T. E. Mallouk , D. Wang , Nat. Mater. 2019, 18, 384. 30858569 10.1038/s41563-019-0305-8

[92] Z. Ju , J. Nai , Y. Wang , T. Liu , J. Zheng , H. Yuan , O. Sheng , C. Jin , W. Zhang , Z. Jin , H. Tian , Y. Liu , X. Tao , Nat. Commun. 2020, 11, 488. 31980618 10.1038/s41467-020-14358-1 PMC6981142

[93] Z. Ju , C. Jin , H. Yuan , T. Yang , O. Sheng , T. Liu , Y. Liu , Y. Wang , F. Ma , W. Zhang , J. Nai , X. Tao , Chem. Eng. J. 2021, 408, 128016.

[94] B. Zhang , H. Shi , Z. Ju , K. Huang , C. Lian , Y. Wang , O. Sheng , J. Zheng , J. Nai , T. Liu , Y. Jin , Y. Liu , C. Zhang , X. Tao , J. Mater. Chem. A 2020, 8, 26045.

[95] Y. Liu , Y. Wu , J. Zheng , Y. Wang , Z. Ju , G. Lu , O. Sheng , J. Nai , T. Liu , W. Zhang , X. Tao , Nano Energy 2021, 82, 105723.

[96] X. Wang , W. Zeng , L. Hong , W. Xu , H. Yang , F. Wang , H. Duan , M. Tang , H. Jiang , Nat. Energy 2018, 3, 227.

[97] Y. Chen , Z. Wang , X. Li , X. Yao , C. Wang , Y. Li , W. Xue , D. Yu , S. Y. Kim , F. Yang , A. Kushima , G. Zhang , H. Huang , N. Wu , Y.-W. Mai , J. B. Goodenough , J. Li , Nature 2020, 578, 251. 32015545 10.1038/s41586-020-1972-y

[98] A. Wang , S. Tang , D. Kong , S. Liu , K. Chiou , L. Zhi , J. Huang , Y. Xia , J. Luo , Adv. Mater. 2018, 30, 1703891. 10.1002/adma.201703891 29125657

[99] Y. Zhang , W. Luo , C. Wang , Y. Li , C. Chen , J. Song , J. Dai , E. M. Hitz , S. Xu , C. Yang , Y. Wang , L. Hu , Proc. Natl. Acad. Sci. USA 2017, 114, 3584. 28320936 10.1073/pnas.1618871114 PMC5389307

[100] G. Li , Z. Liu , Q. Huang , Y. Gao , M. Regula , D. Wang , L.-Q. Chen , D. Wang , Nat. Energy 2018, 3, 1076.

[101] D. Lin , Y. Liu , Z. Liang , H.-W. Lee , J. Sun , H. Wang , K. Yan , J. Xie , Y. Cui , Nat. Nanotechnol. 2016, 11, 626. 26999479 10.1038/nnano.2016.32

[102] C. Jin , O. Sheng , J. Luo , H. Yuan , C. Fang , W. Zhang , H. Huang , Y. Gan , Y. Xia , C. Liang , J. Zhang , X. Tao , Nano Energy, 2017, 37, 177.

[103] C.in. Jin , O. Sheng , Y. Lu , J. Luo , H. Yuan , W. Zhang , H. Huang , Y. Gan , Y. Xia , C. Liang , J. Zhang , X. Tao , Nano Energy 2018, 45, 203. 10.1021/acs.nanolett.8b00659 29692176

[104] L. Liu , Y.-X. Yin , J.-Y. Li , S.-H. Wang , Y.-G. Guo , L.-J. Wan , Adv. Mater. 2018, 30, 1706216. 10.1002/adma.201706216 29334147

[105] H. Yuan , J. Nai , H. Tian , Z. Ju , W. Zhang , Y. Liu , X. Tao , X. W. Lou , Sci. Adv. 2020, 6, eaaz3112. 32181364 10.1126/sciadv.aaz3112 PMC7060059

[106] H. Yuan , J. Nai , Y. Fang , G. Lu , X. Tao , X. W. Lou , Angew. Chem. Int. Ed. 2020, 59, 15839. 10.1002/anie.202001989 32460362

[107] L. Li , S. Basu , Y. Wang , Z. Chen , P. Hundekar , B. Wang , J. Shi , Y. Shi , S. Narayanan , N. Koratkar , Science 2018, 359, 1513. 29599241 10.1126/science.aap8787

[108] X.-G. Yang , G. Zhang , S. Ge , C.-Y. Wang , Proc. Natl. Acad. Sci. 2018, 115, 7266. 29941558 10.1073/pnas.1807115115 PMC6048525

[109] J. Wang , W. Huang , A. Pei , Y. Li , F. Shi , X. Yu , Y. Cui , Nat. Energy 2019, 4, 664.

[110] K. Yan , J. Wang , S. Zhao , D. Zhou , B. Sun , Y. Cui , G. Wang , Angew. Chem. Int. Ed. 2019, 58, 11364. 10.1002/anie.201905251 31148342

[111] A. C. Thenuwara , P. P. Shetty , M. T. McDowell , Nano Lett. 2019, 19, 8664. 31671260 10.1021/acs.nanolett.9b03330

[112] A. C. Thenuwara , P. P. Shetty , N. Kondekar , S. E. Sandoval , K. Cavallaro , R. May , C.-T. Yang , L. E. Marbella , Y. Qi , M. T. McDowell , ACS Energy Lett. 2020, 5, 2411.

[113] J. Holoubek , M. Yu , S. Yu , M. Li , Z. Wu , D. Xia , P. Bhaladhare , M. S. Gonzalez , T. A. Pascal , P. Liu , Z. Chen , ACS Energy Lett. 2020, 5, 1438.

[114] Z. Zhang , J. Yang , W. Huang , H. Wang , W. Zhou , Y. Li , Y. Li , J. Xu , W. Huang , W. Chiu , Yi. Cui , Matter 2021, 4, 302.

[115] X. Xing , Y. Li , X. Wang , V. Petrova , H. Liu , P. Liu , Energy Storage Mater. 2019, 21, 474.

[116] Y. Yang , Y. Yin , D. M. Davies , M. Zhang , M. Mayer , Y. Zhang , E. S. Sablina , S. Wang , J. Z. Lee , O. Borodin , C. S. Rustomji , Y. S. Meng , Energy Environ. Sci. 2020, 13, 2209.

[117] H. Huo , J. Gao , N. Zhao , D. Zhang , N. G. Holmes , X. Li , Y. Sun , J. Fu , R. Li , X. Guo , X. Sun , Nat. Commun. 2021, 12, 176. 33420065 10.1038/s41467-020-20463-y PMC7794502

[118] Y. M. Jeon , S. Kim , M. Lee , W. B. Lee , J. H. Park , Adv. Energy Mater. 2020, 10, 2003114.

[119] M. Hou , F. Liang , K. Chen , Y. Dai , D. Xue , Nanotechnology 2020, 31, 132003. 31770742 10.1088/1361-6528/ab5be7

[120] F. Liang , Y. Sun , Y. Yuan , J. Huang , M. Hou , J. Lu , Mater. Today 2021, 10.1016/j.mattod.2021.03.013.

[121] O. Sheng , C. Jin , J. Luo , H. Yuan , H. Huang , Y. Gan , J. Zhang , Y. Xia , C. Liang , W. Zhang , X. Tao , Nano Lett. 2018, 18, 3104. 29692176 10.1021/acs.nanolett.8b00659

[122] S. Li , S. Zhang , L. Shen , Q. Liu , J. Ma , W. Lv , Y. He , Q. Yang , Adv. Sci. 2020, 7, 1903088. 10.1002/advs.201903088 PMC7055568 32154083

[123] D. Cheng , T. A. Wynn , X. Wang , S. Wang , M. Zhang , R. Shimizu , S. Bai , H. Nguyen , C. Fang , M.-c. Kim , W. Li , B. Lu , S. J. Kim , Y. S. Meng , Joule 2020, 4, 2484.

[124] O. Sheng , J. Zheng , Z. Ju , C. Jin , Y. Wang , M. Chen , J. Nai , T. Liu , W. Zhang , Y. Liu and X. Tao , Adv. Mater. 2020, 32, 2000223. 10.1002/adma.202000223 32656883

[125] O. Sheng , C. Jin , M. Chen , Z. Ju , Y. Liu , Y. Wang , J. Nai , T. Liu , W. Zhang , X. Tao , J. Mater. Chem. A 2020, 8, 13541. 10.1002/adma.202000223 32656883

[126] T. Liu , Jiale. Zheng , H. Hu , O. Sheng , Z. Ju , G. Lu , Y. Liu , J. Nai , Y. Wang , W. Zhang , X. Tao , J. Energy Chem. 2021, 55, 272.

[127] J. Alvarado , M. A. Schroeder , M. Zhang , O. Borodin , E. Gobrogge , M. Olguin , M. S. Ding , M. Gobet , S. Greenbaum , Y. S. Meng , K. Xu , Mater. Today 2018, 21, 341.

